# Chelator Free Radiolabeling and Preclinical Evaluation of η^6^‐Arene Sandwich Complexes of Lenalidomide With Rhenium and Technetium‐99m

**DOI:** 10.1002/cbic.70503

**Published:** 2026-08-21

**Authors:** Qaisar Nadeem, Federica Battistin, Daniel Hernández Valdés, Afaf R. Genady, Luis Rafael Silva Mendez, Saman Sadeghi, Olivier Blacque, Roger Alberto

**Affiliations:** ^1^ International Center for Interdisciplinary Research in Sciences The University of Lahore Lahore Pakistan; ^2^ Department of Chemistry University of Zurich Zurich Switzerland; ^3^ Dipartimento di Scienze Chimiche e Farmaceutiche Università di Trieste Trieste Italy; ^4^ Department of Chemistry and Chemical Biology McMaster University Hamilton Ontario Canada

**Keywords:** lenalidomide, pharmacomimetics, planar chirality, rhenium, sandwich complexes, technetium

## Abstract

Lenalidomide is a clinically important immunomodulatory drug that exerts activity through binding to the intracellular protein cereblon (CRBN). Despite its widespread therapeutic use, radiolabeling strategies that could enable investigation of lenalidomide pharmacokinetics or support the development of metallodrug analogs have not been reported. We report the synthesis and characterization of sandwich complexes [M(η^6^‐lena)_2_]^+^ (M = ^99m^Tc or Re), establishing a bioorganometallic platform for direct, chelator‐free radiolabeling of lenalidomide. [^99m^Tc(η^6^‐lena)_2_]^+^ was synthesized in aqueous media from [^99m^TcO_4_]^−^, while the rhenium analog, [Re(η^6^‐lena)_2_]^+^, enabled structural and biochemical characterization. A monoligand complex, [Re(η^6^‐C_6_H_6_)(η^6^‐lena)]^+^, was synthesized to probe structure–activity relationships, revealing further reduced CRBN binding relative to the bis–ligand complex, [Re(η^6^‐lena)_2_]^+^. While [Re(η^6^‐lena)_2_]^+^ retains measurable binding to CRBN (IC_50_ = 43.8 µM) compared to the parent ligand, lenalidomide (IC_50_ = 7.6 µM). Biodistribution of [^99m^Tc(η^6^‐lena)_2_]^+^ in healthy mice demonstrated rapid blood clearance and predominant renal excretion. These results demonstrate the feasibility of direct radiolabeling of lenalidomide and provide insight into the effects of bioorganometallic modification on CRBN interaction and in vivo pharmacokinetics.

## Graphical abstract

Direct, chelator‐free radiolabeling of lenalidomide is achieved via η^6^‐arene coordination to technetium‐99m and rhenium, forming sandwich complexes. The radiotracer shows rapid blood clearance, predominant renal excretion, and good in vivo stability, highlighting its potential as a pharmacokinetic probe.

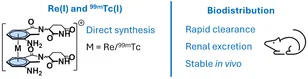

## 1 Introduction

Lenalidomide (Revlimid), a 4‐aminoglutamyl analog of thalidomide, is a clinically important small‐molecule drug widely used for the treatment of multiple myeloma, selected B‐cell malignancies, and myelodysplastic syndrome with del(5q) [1, 2]. The compound exerts its biological effects through interaction with the intracellular protein cereblon (CRBN), a component of the CRL4 E3 ubiquitin ligase complex. Because lenalidomide is a small, membrane‐permeable molecule that distributes systemically and interacts with an intracellular protein, radiolabeled analogs of this scaffold may provide useful molecular tools for investigating the in vivo behavior of immunomodulatory drug (IMiD) derivatives. In particular, minimally modified radiolabeled versions of lenalidomide could enable investigation of pharmacokinetics and tissue distribution while preserving much of the structural framework of the parent drug.

Radiolabeling of small‐molecule drugs with radiometals is commonly achieved through conjugation to bifunctional chelators. However, the introduction of chelating groups often increases molecular size and polarity and can substantially alter physicochemical and biological properties such as membrane permeability and ligand–protein interactions [3, 4]. Chelator‐free strategies based on η^6^‐arene coordination have therefore attracted attention as an alternative approach [5, 13]. In this framework, Tc(I) or Re(I) centers are sandwiched between aromatic ligands to form stable [M(η^6^‐arene)_2_]^+^ complexes (M = ^99m^Tc or Re). These bioorganometallic architectures can be assembled under relatively mild conditions in water and allow direct coordination of aromatic biomolecules without additional conjugation chemistry [14, 17]. Because η^6^‐coordination preserves much of the parent molecule's topology, this strategy provides a route to radiolabeled analogs that retain key structural features of the original drug scaffold.

Beyond radiotracer development, bioorganometallic modification of pharmaceutical agents has also emerged as an approach for generating metallodrug analogs with altered or enhanced biological properties. Incorporation of metal centers into organic drug scaffolds can modulate electronic structure, lipophilicity, and molecular conformation, potentially leading to changes in target interaction or pharmacological activity. In this context, rhenium complexes [Re(η^6^‐arene)_2_]^+^ are particularly valuable as chemically stable homologs that enable the exploration of bioorganometallic derivatives of drug molecules [16, 17]. The preparation of such compounds therefore provides a foundation for evaluating whether metal incorporation into the lenalidomide scaffold can generate new analogs with distinct physicochemical or biological behavior. However, it remains unclear whether direct coordination of lenalidomide to a metal center can preserve its interaction with CRBN while enabling radiotracer development. We hypothesized that η^6^‐coordination would allow direct radiolabeling while partially retaining biological recognition.

Here, we report the chelator‐free η^6^‐coordination‐based radiolabeling of lenalidomide with technetium‐99m together with the synthesis of the corresponding rhenium analogs for structural and biochemical characterization using established methods [14, 16]. These compounds represent the first reported radiometal sandwich complexes derived from lenalidomide. In vitro binding measurements with recombinant CRBN protein were performed to evaluate how η^6^‐coordination affects interaction with the protein scaffold, and biodistribution studies of the technetium complex in healthy mice were conducted to examine the pharmacokinetic behavior of the bioorganometallic derivative. Together, these studies establish a platform for the preparation of lenalidomide‐based radiotracers and provide a basis for exploring bioorganometallic IMiD analogs as potential metallodrug derivatives.

## 2 Results and Discussion

### 2.1 Preparation and Characterization of Rhenium Complexes [Re(η^6^‐lena)_2_]^+^ (1) and [Re(η^6^‐C_6_H_6_)( η^6^‐lena)]^+^ (2)

**FIGURE 1:**
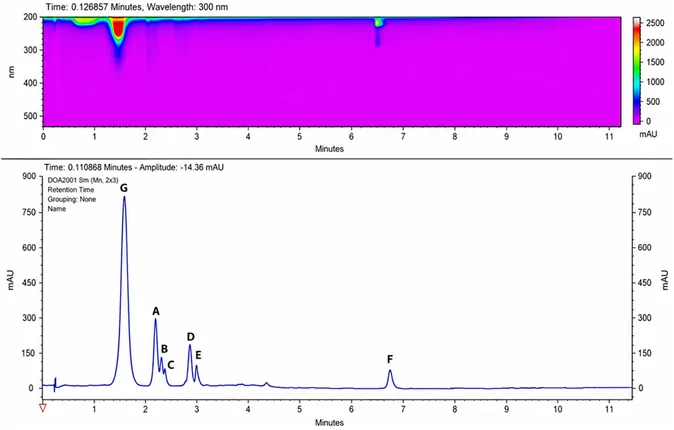
UPLC of the crude product, showing three fractions, A–C, representing mixtures of eight diasteriomeric pairs of [Re(η^6^‐lena)_2_]^+^ (**1**). Fraction G is the ligand (lenalidomide), D,E are [Re(η^6^‐lena)(η^6^‐dihydronaphthalene)]^+^ and [Re(η^6^‐lena)(η^6^‐trihydronaphthalene)]^+^, respectively, and F is free naphthalene.

**SCHEME 1:**
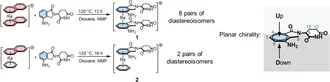
Synthesis of [Re(η^6^‐lena)_2_]^+^ (**1**) and [Re(η^6^‐C_6_H_6_)(η^6^‐lena)]^+^ (**2**) (left) and lenalidomide with the numbering scheme showing the planar chirality (right). All stereochemical configurations are illustrated in Figures S1 and 4 for [Re(η^6^‐lena)_2_]^+^ and [Re(η^6^‐C_6_H_6_)(η^6^‐lena)]^+^, respectively.

The complex [Re(η^6^‐lena)_2_]TFA (**1TFA**) was synthesized along a previously reported procedure [16] based on the arene substitution of naphthalene in [Re(η^6^‐napht)_2_]PF_6_ by lenalidomide (Scheme 1). The starting material [Re(η^6^‐napht)_2_]PF_6_ was synthesized according to a reported procedure [15]. A solution of [Re(η^6^‐napht)_2_]PF_6_ in pure NMP was added dropwise over approximately 6.5 h to a heated solution of lenalidomide in NMP/dioxane, followed by heating at 120 °C for 12 h. The ultra performance liquid chromatography (UPLC) of the crude reaction solution showed at least three different fractions, A, B, and C, representing mixtures of eight disteriomeric pairs of [Re(η^6^‐lena)_2_]^+^ characterized by UPLC‐mass spectrometry (MS) (Figure 1), which could be separated by preparative high performance liquid chromatography (HPLC). The isolated yields of the three fractions A, B, and C, were 20%, 6.4%, and 7%, respectively (overall 33%).

With asymmetrically substituted arenes or arenes comprising a chirality center in the pendent group, binding can occur from two “nonequivalent” sides (designated here as **U**p/**D**own in Scheme 1) [16]. When coordinated in an η^6^ manner to a metal forming sandwich complexes, planar chirality occurs in the final complexes [18, 20]. Such planar chiral features are common in ferrocene [21, 23] and Ru–arene [24] sandwich systems but remain rare in rhenium chemistry [25]. Thus, each coordinated lenalidomide introduces both central (R/S) and planar (**U**/**D**) chirality. In principle, the combination of two stereogenic centers and two planar chiral elements thus gives rise to 16 distinct stereochemical configurations (8 enantiomeric pairs) for [Re(η^6^‐lena)_2_]^+^. The stereochemical assignments for all eight diastereomeric pairs are presented in Figure S1.

**FIGURE 2:**
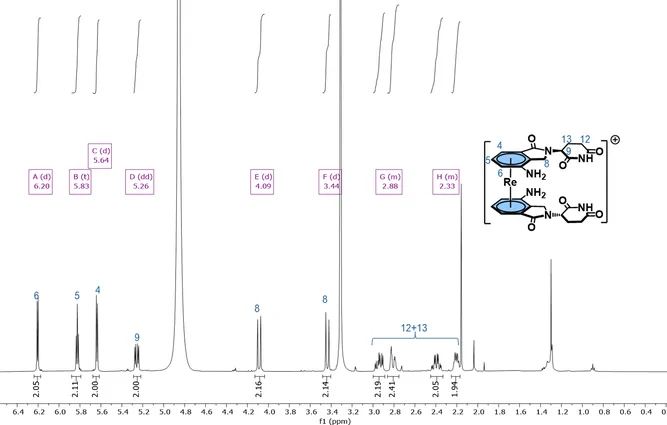
^1^H NMR spectrum in MeOD of fraction C, [Re(η^6^‐lena)_2_]^+^ (**1**), enantiomeric pair (**U**,*R;* **U**,*R*) and (**D**,*S;* **D**,*S*).

Preparative HPLC separation gave three fractions (A–C). According to the ^1^H NMR spectra in CD_3_OD, fractions A and B consisted of mixtures of diastereomers, whereas fraction C contains one single diastereoisomer of enantiomers. Since NMR spectroscopy cannot differentiate between enantiomers, fraction C appears as a single pure compound in solution (numbering scheme is shown in Scheme 1). In the ^1^H NMR spectrum of fraction C, the η^6^‐coordinated aromatic protons of lenalidomide appear as two doublets at *δ* 6.20 (H6) and 5.64 (H4) ppm and a triplet at *δ* 5.83 ppm (H5) (Figure 2), consistent with η^6^‐arene coordination to the rhenium center. Notably, coordination induces pronounced changes in the aliphatic region. In free lenalidomide, the methylene protons of the isoindolinone ring (H8) are magnetically equivalent and resonate as a doublet at *δ* 4.30 ppm with a coupling constant of 4.1 Hz due to nearest chiral proton present. Upon coordination in [Re(η^6^‐lena)_2_]^+^ (fraction C), these methylene protons become diastereotopic due to the rigid chiral environment generated by both the metal‐induced planar chirality and the central chirality of the ligand. They appear as two doublets at *δ* 4.09 and 3.44 ppm with a geminal coupling constant of 14.8 Hz. The proton at the stereogenic carbon within the piperidinedione ring of free ligand appears as multiplet at 5.16 ppm, and it was same in the Re complex at *δ* 5.26 ppm, arising from coupling with the adjacent nonequivalent methylene protons (H13a and H13b) and also with the methylene protons of the isoindolinone ring (H8a and H8b). The remaining piperidine methylene protons (H12 and H13) resonate as multiplets over *δ* 2.94–2.20 ppm. These spectroscopic features are fully consistent with a conformationally rigid, doubly chiral coordination environment.

In the ^1^H NMR spectrum of fraction B (Figures S4, S5), two diastereoisomers are observed in approximately a 1:1 ratio. The η^6^‐coordinated aromatic protons of lenalidomide display duplicated resonances consistent with the presence of two stereoisomeric species. In particular, the H6 protons appear as two closely spaced doublets at *δ* 6.20 and 6.17 ppm, while the H5 proton is observed as a multiplet centered at *δ* 5.80 ppm, for both diastereoisomers; the H4 protons give rise to two distinct doublets at *δ* 5.67 and 5.64 ppm. The proton at the stereogenic carbon of the piperidinedione ring (H9) is clearly duplicated, appearing at *δ* 5.23 and 4.67 ppm for the two diastereoisomers. The methylene protons of the isoindolinone ring (H8) are diastereotopic and appear as two pairs of geminally coupled doublets: one pair at *δ* 4.18 and 3.67 ppm (*J* = 15.1 Hz) and a second pair at *δ* 4.07 and 3.42 ppm (*J* = 14.8 Hz). The remaining piperidine methylene protons (H12 and H13) resonate as overlapping multiplets in the region *δ* 3.00–2.10 ppm.

The ^1^H NMR spectrum of fraction A indicates the presence of a more complex mixture, consistent with at least four diastereoisomers in solution (Figures S6–S9). This is most clearly evidenced by the proton at the stereogenic carbon of the piperidinedione ring (H9), which gives rise to at least three well‐resolved multiplets at *δ* 5.24, 5.18, and 4.69 ppm, together with a fourth signal partially obscured by the residual water resonance in CD_3_OD. In the aromatic region, the overlap of signals prevents a straightforward distinction of individual diastereomeric patterns in the ^1^H spectrum alone. However, ^1^H–^13^C heteronuclear single quantum correlation (HSQC) (Figure S9) correlations allow the identification of four distinct H4 resonances, each correlating with a different carbon signal in the range *δ* *C* ≈ 60–66 ppm. The corresponding C5 and C6 carbons are observed in the range *δ* *C* ≈ 70–80 ppm, consistent with η^6^ coordination to the rhenium center and in agreement with the assignments made for fractions B and C. The methylene protons of the isoindolinone ring (H8) appear as a series of overlapping multiplets at *δ* 4.12, 3.89, 3.80, and 3.66 ppm, consistent with multiple diastereomeric environments, although individual geminal couplings cannot be unambiguously resolved. The remaining piperidine methylene protons (H12 and H13) resonate as multiplets in the region *δ* 3.00–2.00 ppm. Overall, the extensive signal duplication and overlap observed in both the aromatic and aliphatic regions are fully consistent with the presence of multiple diastereoisomers of [Re(η^6^‐lena)_2_]^+^ in fraction A.

The ^1^H NMR analysis of fractions A–C highlights the pronounced stereochemical complexity of [Re(η^6^‐lena)_2_]^+^ in solution. Fraction A displays the highest spectral complexity and is consistent with the presence of at least four diastereoisomers; fraction B contains two diastereoisomers in approximately equal proportions; fraction C corresponds to one single diastereoisomer. Overall, the combined analyses of fractions A–C indicate that seven of the eight stereochemically possible isomers of [Re(η^6^‐lena)_2_]^+^ are observed experimentally, underscoring the rich stereochemical landscape generated by the interplay of planar and central chirality in these η^6^‐arene rhenium complexes.

**FIGURE 3:**
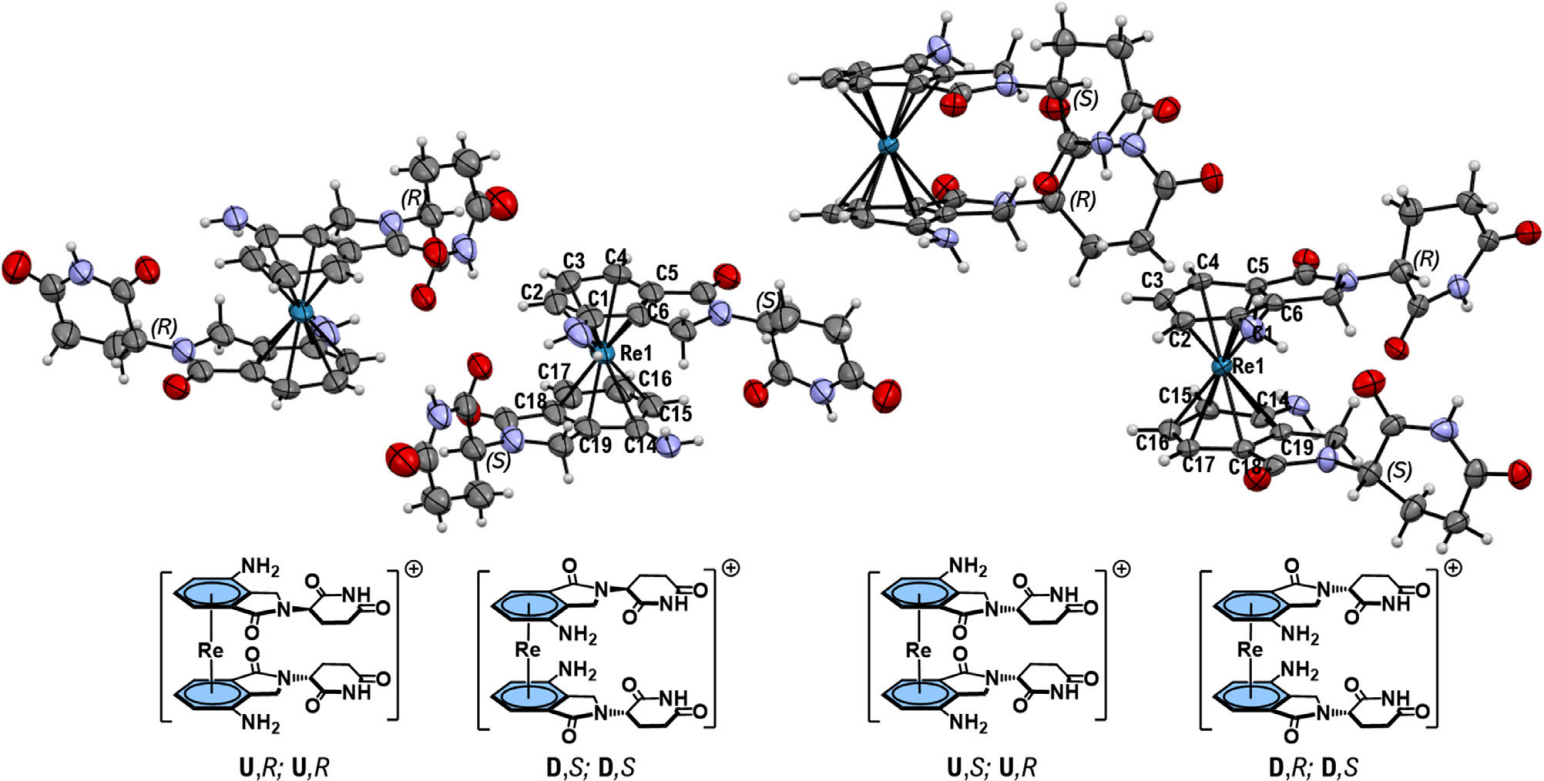
Ellipsoid displacement plots of [Re(η^6^‐lena)_2_]^+^ (**1**) of fraction C, enantiomeric pair (**U**,*R;* **U**,*R*) and (**D**,*S;* **D**,*S*) (left) and in fraction B, enantiomeric pair (**U**,*S;* **U**,*R*) and (**D**,*R;* **D**,*S*) (right). Ellipsoids are drawn at 50% probability. The anions (TFA for fraction C and TFA + [ReO_4_]^−^ for fraction B) are omitted for clarity. Coordination distances in Ångström (Å): enantiomeric pair (**U**,*R;* **U**,*R*) and (**D**,*S;* **D**,*S*): Re1‐C1 = 2.368(9), Re1‐C2 = 2.243(9), Re1‐C3 = 2.218(10), Re1‐C4 = 2.285(9), Re1‐C5 = 2.231(9), Re1‐C6 = 2.233(6), Re1‐C14 = 2.355(10), Re1‐C15 = 2.231(9), Re1‐C16 = 2.228(9), Re1‐C17 = 2.274(10); Re1‐C18 = 2.235(8), Re1‐C19 = 2.246(9); enantiomeric pair (**U**,*S;* **U**,*R*) and (**D**,*R;* **D**,*S*): Re1‐C1 = 2.375(16), Re1‐C2 = 2.201(15), Re1‐C3 = 2.22(2), Re1‐C4 = 2.280(15), Re1‐C5 = 2.197(15), Re1‐C6 = 2.267(17), Re1‐C14 = 2.386(15), Re1‐C15 = 2.183(17), Re1‐C16 = 2.24(2), Re1‐C17 = 2.253(15); Re1‐C18 = 2.227(16), Re1‐C19 = 2.264(16).

Single‐crystal X‐ray diffraction of the single diastereomer from fraction C could be elucidated. The compound crystallizes in the centrosymmetric space group P–1 (Figure 3 left). Due to the inversion symmetry, each molecule in the asymmetric unit is accompanied by its inversion‐related enantiomer within the unit cell. Accordingly, the diastereoisomer present in fraction C crystallizes as a racemic pair, and its absolute configuration can be described as (**U**,*R;* **U**,*R*) and (**D**,*S;* **D**,*S*).

Crystals obtained from fraction B, which contains two diastereoisomers in solution, correspond to only one diastereoisomer of the complex. This compound crystallizes in the noncentrosymmetric, chiral space group Pca2_1_ (Figure 3 right). As Pca2_1_ lacks inversion symmetry, the asymmetric unit contains a single enantiomer; the corresponding enantiomeric partner is generated by the crystallographic symmetry operations of the unit cell. Thus, although fraction B consists of a mixture of diastereoisomers in solution, crystallization selectively yields one diastereoisomer, present in the solid state as the enantiomeric pair (**U**,*S;* **U**,*R*) and (**D**,*R;* **D**,*S*) (Figure 3 right).

**FIGURE 4:**
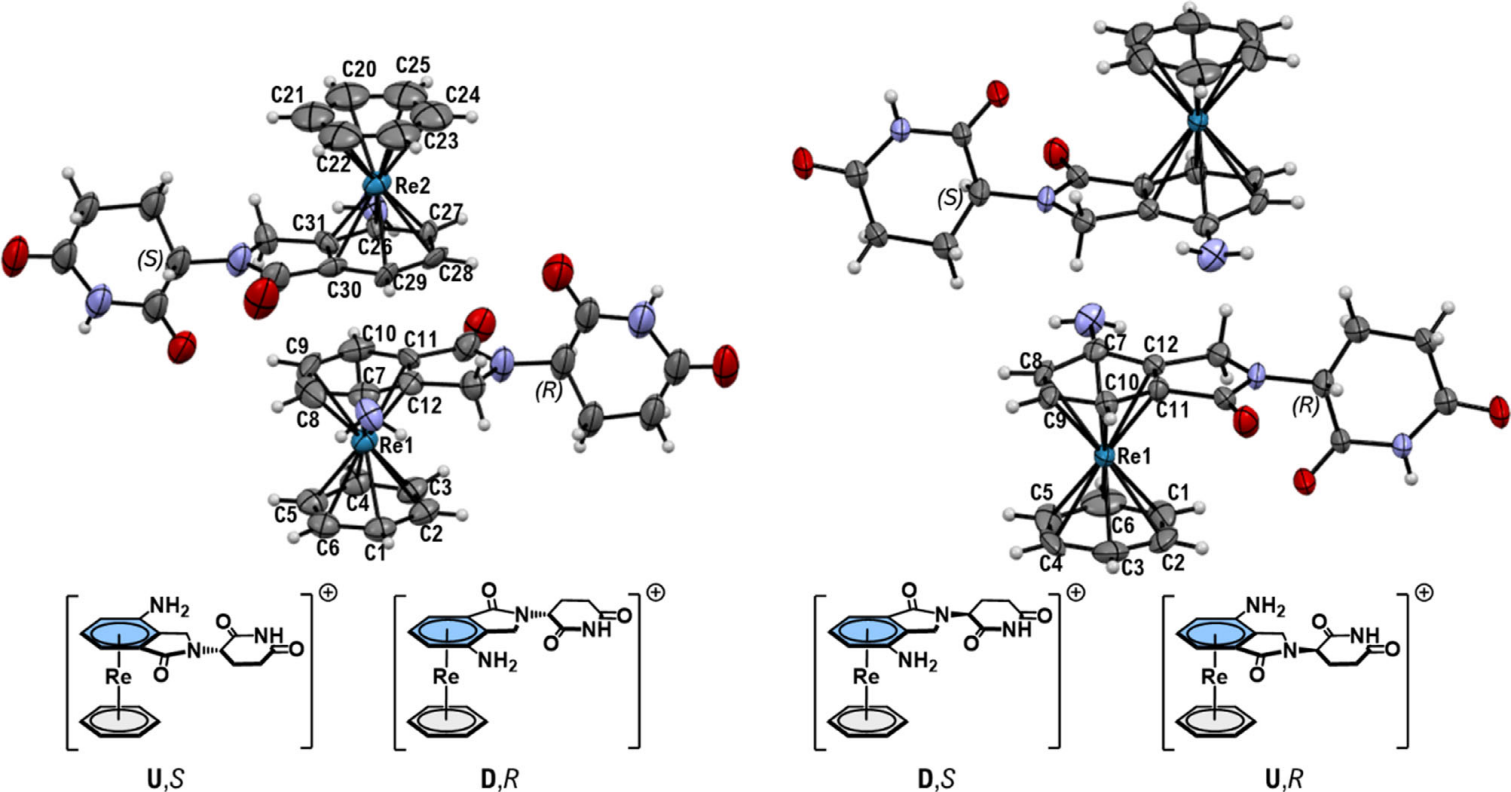
Ellipsoid displacement plots of [Re(η^6^‐C_6_H_6_)(η^6^‐lena)]^+^ (**2**) enantiomeric pair (**U**,*S*) and (**D**,*R*) (left) and enantiomeric pair (**U**,*R*) and (**D**,*S*) (right). Ellipsoids are drawn at 50% probability. The TFA^**−**^ anions are omitted for clarity. Coordination distances in Ångström (Å): enantiomer (**D**,*R*): Re1‐C1 = 2.207(12), Re1‐C2 = 2.216(12), Re1‐C3 = 2.253(12), Re1‐C4 = 2.217(12), Re1‐C5 = 2.224(12), Re1‐C6 = 2.220(13), Re1‐C7 = 2.350(11), Re1‐C8 = 2.249(15), Re1‐C9 = 2.199(12), Re1‐C10 = 2.229(11); Re1‐C11 = 2.210(11), Re1‐C12 = 2.215(11); enantiomer (**U**,*S*): Re2‐C20 = 2.209(18), Re2‐C21 = 2.265(19), Re2‐C22 = 2.207(17), Re2‐C23 = 2.200(17), Re2‐C24 = 2.195(19), Re2‐C25 = 2.206(19), Re2‐C26 = 2.352(11), Re2‐C27 = 2.211(12), Re2‐C28 = 2.242(10), Re2‐C29 = 2.251(10); Re2‐C30 = 2.243(10), Re2‐C31 = 2.258(12); enantiomeric pair (**U**,*R*) and (**D**,*S*): Re1‐C1 = 2.241(8), Re1‐C2 = 2.242(8), Re1‐C3 = 2.210(9), Re1‐C4 = 2.219(8), Re1‐C5 = 2.227(8), Re1‐C6 = 2.201(9), Re1‐C7 = 2.395(7), Re1‐C8 = 2.225(7), Re1‐C9 = 2.218(7), Re1‐C10 = 2.250(7); Re1‐C11 = 2.220(7), Re1‐C12 = 2.234(7).

For subsequent biological studies, we prepared the complex [Re(η^6^‐C_6_H_6_)(η^6^‐lena)]TFA from reaction of [Re(η^6^‐C_6_H_6_)(η^6^‐napht)]PF_6_ and lenalidomide following a reported procedure [26]. The complex [Re(η^6^‐C_6_H_6_)(η^6^‐lena)]^+^ was obtained in 58% yield after HPLC purification. Single‐crystal X‐ray diffraction revealed two distinct solid‐state forms of [Re(η^6^‐C_6_H_6_)(η^6^‐lena)]^+^ (Figure 4). The first form crystallizes in the noncentrosymmetric, chiral space group P2_1_ and was refined as a two‐component inversion twin, with nearly equal twin fractions. This clearly indicated that the crystal is a racemic twin, consisting of two intergrown domains related by inversion. The two crystallographically independent molecules therefore correspond to the enantiomeric pair (**U**,*S*) and (**D**,*R*), even though only one enantiomer is present in each individual domain.

The second crystal form crystallizes in the centrosymmetric space group P2_1_/n, which contains an inversion center in the asymmetric unit. As expected for a centrosymmetric space group, the complex is present as an inversion‐related racemic pair, corresponding to the enantiomers (**U**,*R*) and (**D**,*S*). This crystal form therefore represents the second enantiomeric pair accessible for this system.

Together, the P2_1_ inversion‐twin crystal ((**U**,*S*)/(**D**,*R*)) and the P2_1_/n centrosymmetric crystal ((**U**,*R*)/(**D**,*S*)) account for all four possible stereochemical configurations of the complex.

The ^1^H NMR spectrum of [Re(η^6^‐C_6_H_6_)(η^6^‐lena)]^+^ in CD_3_OD (Figure S10) clearly indicates the presence of two distinct diastereoisomers in solution, in an approximate 1:4 ratio. Resonance assignments were established by ^1^H–^1^H COSY and ^1^H–^13^C HSQC analyses (Figures S12 and S13), using the numbering scheme shown in Scheme 1. The η^6^‐coordinated benzene ligand appears as two singlets at *δ* 5.79 and 5.77 ppm in a 1:4 ratio, corresponding to the two diastereoisomeric forms. The aromatic protons of the coordinated lenalidomide ring give four additional signals: *δ* 6.37 (H6, minor diastereoisomer), *δ* 6.34 (H6, major diastereoisomer), *δ* 6.08 (H5, overlapping resonances of both diastereoisomers), and *δ* 5.82 ppm (H4, both diastereoisomers). The methylene protons of the isoindolinone ring (H7) resonate as signals centered at *δ* 4.19 ppm; although their diastereotopic character is apparent, the individual components are only partially resolved. The proton at the stereogenic carbon of the piperidinedione ring (H9) appears as two well‐separated multiplets at *δ* 5.21 and 4.69 ppm, corresponding to the minor and major diastereoisomer, respectively. The remaining piperidine methylene protons (H12 and H13) appear as multiplets over the region *δ* 2.95–2.10 ppm. Overall, the duplication of selected resonances with unequal intensities—together with the characteristic η^6^‐benzene signal—is fully consistent with the presence of two conformationally rigid diastereomeric forms of [Re(η^6^‐C_6_H_6_)(η^6^‐lena)]^+^ in solution.

### 2.2 Preparation and Characterization of Technetium Complex ^99m^Tc(η^6^‐lena)_2_]^+^ (3)

**SCHEME 2:**
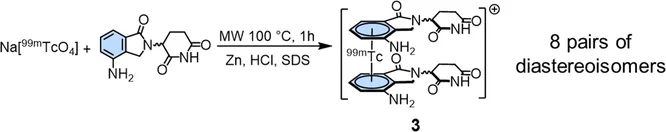
Synthesis of [^99m^Tc(η^6^‐lenalidomide)_2_]^+^ **(3)**.

The [^99m^Tc(η^6^‐lenalidomide)_2_]^+^ (3) complex was synthesized directly in aqueous media following our previously established methodology as given in Scheme 2 [14, 16]. The [^99m^Tc(η^6^‐lenalidomide)_2_]^+^ complex was obtained in approximately 10%–30% radiochemical yield via the direct reaction of generator‐eluted [^99m^TcO_4_]^−^, pertechnetate with lenalidomide in the presence of elemental zinc under acidic conditions. Yield was improved from 10% (with Zn‐powder) to 30% (with zinc turning). Zn/HCl acted as the reducing agent. The reaction mechanism in highly complex reaction scheme may involve many steps, which can only be speculated. Briefly, it involves total 6 e^−^ reduction from Tc^VII^ to Tc^I^, transfer of 8 H^+^ to form 4 H_2_O molecules, and two lenalidomide ligands are coordinated to reduced Tc^I^ center.

**FIGURE 5:**
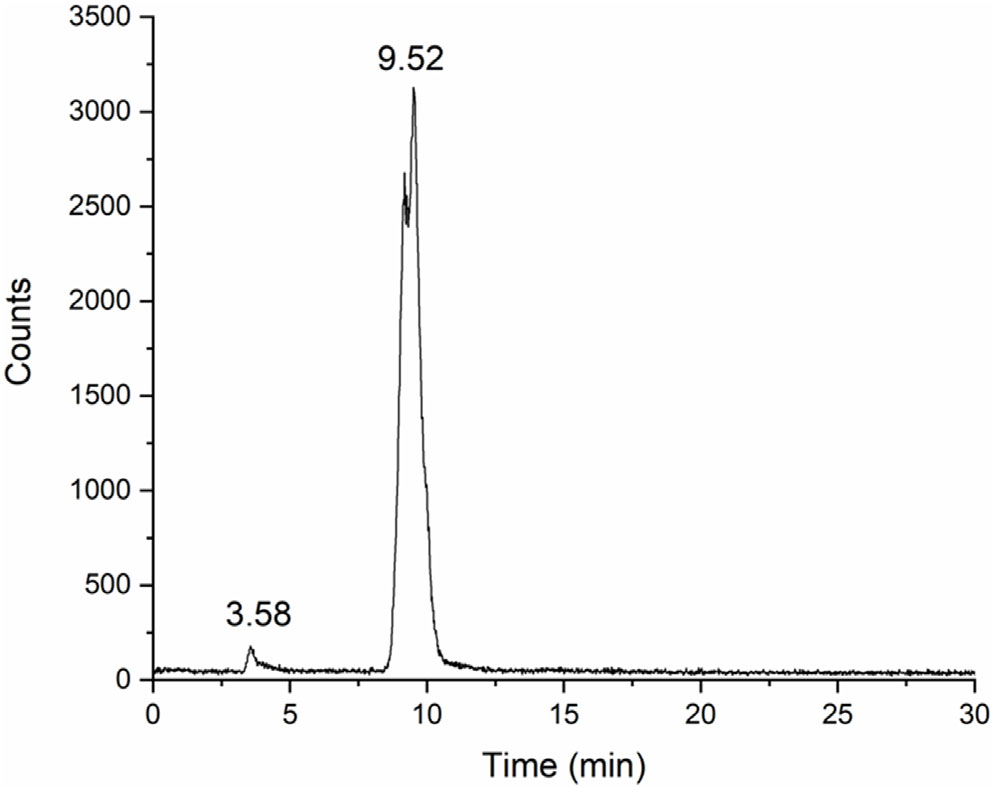
Raw radio‐chromatogram of [^99m^Tc(η^6^‐lena)_2_]^+^, peaks at 9.52 and 3.58 min representing eight possible diastereoisomers pairs of [^99m^Tc(η^6^‐lena)_2_]^+^ and [^99m^TcO_4_]^−^, respectively.

Figure 5 shows the raw radio‐HPLC chromatogram of the crude reaction solution using gradient G3 (given in the Supporting Information), resulting in the desired [^99m^Tc(η^6^‐lenalidomide)_2_]^+^ trace at 9.52 min with 98% radiochemical purity and very less unreacted starting material, [^99m^TcO_4_]^−^ (2%) at 3.58 min. It shows that no side product was formed. The trace at 9.52 min containing all eight possible diastereoisomers pairs of [^99m^Tc(η^6^‐lenalidomide)_2_]^+^ was collected and mixed with its Re analogues diastereoisomers pairs of [Re(η^6^‐lenalidomide)_2_]^+^ for co‐injection.

**FIGURE 6:**
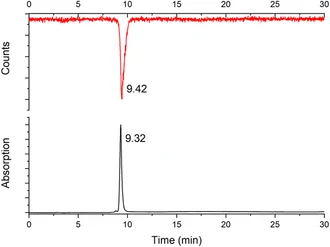
Co‐injection of [^99m^Tc(η^6^‐lena)_2_]^+^ (**3**) (top) and [Re(η^6^‐lena)_2_]^+^ (**1**) (bottom).

**FIGURE 7:**
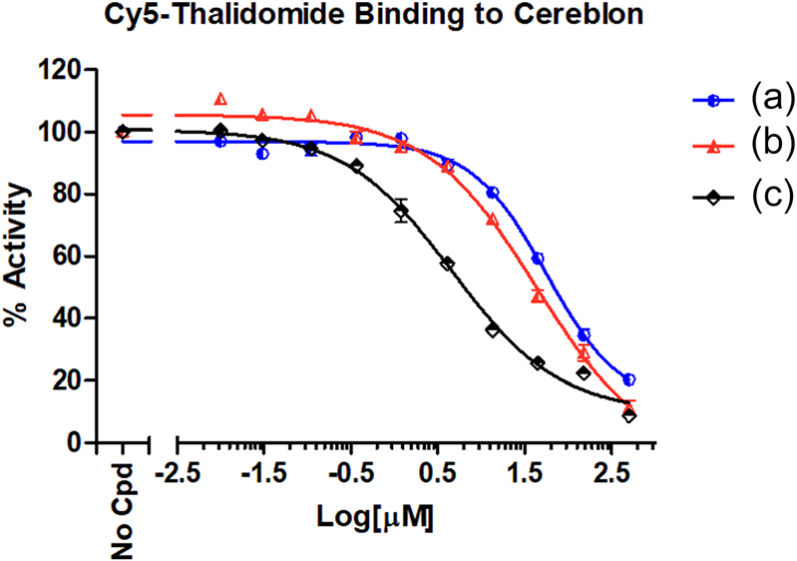
In vitro Cy5‐Thalidomide binding to CRBN. Changes in fluorescence polarization are measured at different concentrations of (a) [Re(η^6^‐C_6_H_6_)(η^6^‐lena)]^+^ (2), (b) [Re(η^6^‐lena)_2_]^+^ (1), and (c) lenalidomide, which are used as competing ligands to determine IC_50_ values. Data are presented as the average of three replicates ± SD.

The identity of the resulting complex was confirmed by comparing the HPLC retention time of the rhenium homolog by co‐injection (Figure 6) using HPLC gradient G3. The [^99m^Tc(η^6^‐lenalidomide)_2_]^+^ (3) complex is expected to exist as a mixture of stereoisomers analogous to the rhenium system. Due to short half‐life of ^99m^Tc, no stereochemical separation was performed. Although no separate stability studies were conducted for [^99m^Tc(η^6^‐lenalidomide)_2_]^+^, previous studies [14, 16] showed the complexes of the type [^99m^Tc(η^6^‐arene)_2_]^+^ are very stable over a wide range of pH values and temperatures (up to 160 °C) and remained stable when solutions were kept in air overnight. Figure 7 reveals low stomach uptake (2.05 ± 0.32 and 1.02 ± 0.09%ID/g at 30 and 90 min, respectively) further indicating minimal in vivo dissociation or re‐oxidation to free [^99^ ^m^TcO_4_]^−^ anion.

### 2.3 Biological Studies

#### 2.3.1 Recombinant CRBN Protein Binding of Rhenium Complexes

**FIGURE 8:**
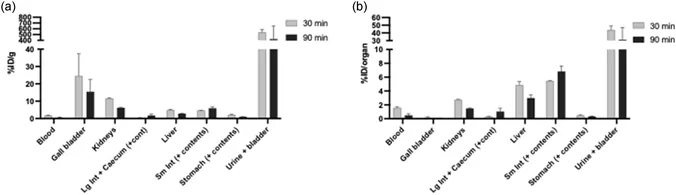
Biodistribution studies of [^99m^Tc(η^6^‐lena)_2_]^+^ (3) formulated in 10% ethanol in saline in Balb/c mice. Two time points were evaluated, 30 and 90 min (*n* = 3). (a) %ID/g and (b) %ID/organ.

The crystal structure of human Cereblon‐DDB1 with bound lenalidomide in the literature [1] showed that lenalidomide binds CRBN via a conserved hydrophobic tri‐tryptophan pocket (Trp380, Trp386, Trp400) that tightly accommodates the glutarimide moiety through hydrophobic contacts and key hydrogen bonds, while the isoindolinone ring remains solvent‐exposed. Mutations in this pocket stop drug binding and its anticancer activity, showing this site is essential for function. In this study, the binding affinities of [Re(η^6^‐lena)_2_]^+^, [Re(η^6^‐C_6_H_6_)(η^6^‐lena)]^+^, lenalidomide, and the reference compound pomalidomide toward CRBN were evaluated using an in vitro competitive fluorescence polarization assay. The rhenium complexes exist as mixtures of stereoisomers arising from combined central and planar chirality. No separation of individual enantiomers was performed for the biological studies, and the reported binding affinities therefore reflect the behavior of these stereoisomeric mixtures. Cy5‐labeled thalidomide was first bound to CRBN, and displacement by increasing concentrations of the test compounds was monitored (Tables S3–S7). Fluorescence polarization data were converted to percentage activity and plotted against compound concentration. Nonlinear regression using a sigmoidal dose–response model generated binding curves (Figure 8), from which IC_50_ values, representing the concentration required for 50% inhibition, were determined.

As summarized in Table 1, pomalidomide exhibited the highest binding affinity, with an IC_50_ of 3.0 µM, followed by lenalidomide (IC_50_ = 7.6 µM). The higher affinity of pomalidomide may be attributed to its chemical modifications relative to lenalidomide, which enhance hydrophobic contacts and hydrogen bonding interactions within the CRBN binding pocket. These modifications likely improve positioning of the glutarimide moiety and strengthen overall ligand–protein interactions. The rhenium sandwich complex [Re(η^6^‐lena)_2_]^+^ showed reduced binding (IC_50_ = 43.8 µM), approximately sevenfold higher IC_50_ than free lenalidomide. This decrease in affinity is likely due to steric hindrance, as the glutarimide moiety cannot optimally fit into the CRBN hydrophobic pocket when two ligands are symmetrically coordinated, indicating that such coordination does not enhance CRBN binding.

**TABLE 1:** Summary of inhibitory effects of all tested compounds on CRBN activity.

Compound	**IC**_**50,**_ **µM**	95% C.I., µM
[Re(η^6^‐C_6_H_6_)(η^6^‐lena)]TFA	~74	61.2–85.5
[Re(η^6^‐lena)_2_]TFA	43.8	35.6–52.5
Lenalidomide	7.6	4.9–9.6
Pomalidomide	3.0	2.7–3.4

The mixed–ligand complex [Re(η^6^‐C_6_H_6_)(η^6^‐lena)]^+^ exhibited slightly lower affinity than [Re(η^6^‐lena)_2_]^+^, with an IC_50_ of approximately 74 µM. The further loss of affinity in [Re(η^6^‐C_6_H_6_)(η^6^‐lena)]^+^ (2), despite its reduced steric bulk, can be explained by constrained ligand orientation imposed by rhenium coordination. In this complex, the glutarimide moiety may not fully engage the CRBN pocket, while the benzene ligand provides minimal stabilizing interactions and may partially obstruct binding. Additionally, the positively charged rhenium center could weaken hydrogen bonding and van der Waals contacts. Collectively, these results emphasize that binding in such sandwich complexes is influenced not only by sterics but also by ligand orientation, electronic effects, and cooperative interactions between coordinated ligands.

#### 2.3.2 Biodistribution Studies of [^99m^Tc(η^6^‐lena)_2_]^+^ (3)

Biodistribution studies of mixtures of stereoisomers of [^99m^Tc(η^6^‐lena)_2_]^+^ (3) were conducted in two cohorts of healthy mice (*n* = 3 per group) at 30 and 90 min postinjection, as summarized in Figure 7a,b, Tables S8 and S9. The in vivo profile is characterized by rapid blood clearance of the radiotracer, with activity levels decreasing from 1.71 ± 0.14%ID/g at 30 min to 0.52 ± 0.26%ID/g at 90 min (Figure 7a). Predominant rapid renal excretion was observed, as evidenced by the majority of the radioactivity being detected in the urine at 30 min and kidney uptake values of 11.55 ± 0.33 and 6.16 ± 0.35%ID/g at 30 and 90 min, respectively. Some hepatobiliary excretion was also observed, with moderate uptake observed in the liver (4.81 ± 0.36 and 2.82 ± 0.32%ID/g) and small intestine (4.64 ± 0.15 and 5.88 ± 0.88%ID/g) at 30 and 90 min postinjection, respectively. The slight increase in intestinal activity at 90 min is attributed to delayed biliary transit. Relatively higher gallbladder uptake (24.49 ± 12.81 and 15.47 ± 7.10%ID/g at 30 and 90 min, respectively) likely reflects concentration of the radiotracer in bile and the small mass of this organ, rather than a major contribution to overall hepatobiliary clearance. The dominance of renal over hepatobiliary elimination suggests that the radiotracer possesses increased hydrophilicity and limited lipophilic character. Low stomach uptake (2.05 ± 0.32 and 1.02 ± 0.09%ID/g at 30 and 90 min, respectively) further indicates minimal in vivo dissociation or re‐oxidation to free [^99m^TcO_4_]^−^ pertechnetate.

## 3 Conclusion

In this study, we synthesized chelator‐free η^6^‐arene sandwich complexes of lenalidomide with technetium‐99m and rhenium, [M(η^6^‐lena)_2_]^+^ (M = ^99m^Tc, Re), representing the first radiometal‐derived complex based on the lenalidomide scaffold. These complexes adopt well‐defined η^6^‐arene sandwich geometries and constitute uncommon examples of rhenium and technetium systems displaying coexisting central and planar chirality.

Although the rhenium complexes [Re(η^6^‐lena)_2_]^+^ and [Re(η^6^‐C_6_H_6_)(η^6^‐lena)]^+^ exhibited lower binding affinity compared to the parent ligand, measurable interaction with CRBN was still observed, indicating that η^6^‐ coordination compromises but does not abolish CRBN recognition. Biodistribution studies of the technetium‐99m complex [^99m^Tc(η^6^‐lena)_2_]^+^ in healthy mice revealed rapid blood clearance and predominant renal excretion, consistent with the small‐molecule character of the scaffold and indicating good in vivo stability of the bioorganometallic complex. These results demonstrate the feasibility of direct, chelator‐free radiolabeling of lenalidomide using η^6^‐coordination chemistry while largely preserving the physicochemical characteristics of the parent drug framework.

The radiolabeling reaction in this study was intended for ^99m^Tc. As half‐life of ^99m^Tc is 6.01 h, the method is just sufficient for ^99m^Tc. Considering the slow kinetics, 1 h reaction time, this method limits for radioisotope having shorter half‐life in range of 1–2 h or less. The naphthalene exchange reactions for incoming ligand during the formation of [Re(η^6^‐lena)_2_]^+^ and [Re(η^6^‐C_6_H_6_)(η^6^‐lena)]^+^ were carried out at 120 °C. Such reaction conditions may not be suitable for temperature sensitive biomolecules indeed.

More broadly, this work expands the chemical space of IMiD‐derived metal complexes and illustrates how bioorganometallic modification can be applied to established pharmaceutical scaffolds. The corresponding rhenium analogs provide chemically stable homologs suitable for further exploration of metallated lenalidomide derivatives, while the technetium complex offers a convenient radiotracer platform for probing the pharmacokinetic behavior of this drug class in vivo. The future direction is to develop single‐isomer‐based labeling to potentiate the downstream process instead of mixtures of many isomers. Some of the isomers may be inactive and as a result this will lead to decreased binding affinity, overall. This is due to the fact that we consider the total weight of all isomeric mixtures including active and inactive isomers in concentration calculation for IC_50_.

## 4 Experimental Sections

### 4.1 Materials and Methods

Details are given in Supporting Information for this section.

### 4.2 Synthesis of [Re(η^6^‐lena)_2_]TFA (1TFA)

**Solution A:** [Re(η^6^‐naphthalene)_2_]PF_6_ (52.88 mg, 0.09 mmol, 1 equiv) was dissolved in dry *N*‐methyl‐2‐pyrrolidone (NMP, 250 µL) to afford a red solution. The solution was transferred to a 250 µL glass Hamilton syringe and mounted on an automated syringe pump. **Solution B:** A two‐necked Schlenk tube was charged with lenalidomide (583.34 mg, 2.25 mmol, 25 equiv), dry NMP (800 µL), and dry dioxane (1.4 mL) under a nitrogen atmosphere. The mixture was equipped with a magnetic stirrer and a reflux condenser and heated to 120 °C, forming an orange suspension. Solution A was added dropwise to Solution B at a constant flow rate of 40 µL h^−1^. Within approximately 10 min, the orange suspension gradually turned light brown after the initial drops were added. Complete addition of Solution A required 6.5 h, resulting in a dark brown solution. The reaction mixture was then maintained at 120 °C with stirring for a total reaction time of 12 h (from the start of addition), yielding a brownish suspension. After cooling to room temperature, NMP and dioxane were removed in vacuo at 80 °C. The resulting brown crude residue was washed with dry diethyl ether (1.5 mL × 5). The residue was then extracted with acetone (2 mL × 4), and the combined acetone extracts were filtered through a glass frit to give a brown filtrate. Removal of the solvent under reduced pressure at 50 °C afforded a brownish crude product. The crude material was dissolved in 4.5 mL of a 50% (v/v) acetonitrile in water solution containing trifluoroacetic acid (0.1%), filtered, and purified by preparative HPLC using gradient G1 (given in Supporting Information). Three fractions (A–C) were collected separately and freeze‐dried to afford [Re(η^6^‐lena)_2_]TFA as a yellow solid. Yield of fraction A, B, and C are (14.8 mg) 20.11%, (4.75 mg) 6.4% and (5.19 mg) 7% respectively, (overall 33.51%). Single crystals suitable for X‐ray diffraction were obtained from fractions B and C, by vapor diffusion from Et_2_O (antisolvent) into CH_3_CN (solvent).**^1^H NMR** (500 MHz, CD_3_OD) fraction A (mixture of at least four diastereoisomers): *δ* 6.24–6.13 (m, 4H), 6.06 (td, *J* = 9.2, 5.1 Hz, 4H), 5.92 (m, 6H), 5.86–5.72 (m, 6H), 5.21 (m, 4H), 4.70 (m, 1H), 4.23–4.04 (m, 7H), 3.94–3.56 (m, 7H), 3.03–2.70 (m, 16H), 2.57–2.14 (m, 16H). **^13^C NMR** (125 MHz, CD_3_OD) fraction A (mixture of at least four diastereoisomers): *δ* 174.54 (C=O), 174.39 (C=O), 174.33 (C=O), 172.58 (C=O), 172.38 (C=O), 172.24 (C=O), 172.16 (C=O), 172.02 (C=O), 171.27 (C=O), 123.21 (C1), 122.61 (C1), 121.68 (C1), 81.94 (C2), 80.76 (C2), 80.42 (C2), 79.64 (C2), 79.32 (C2), 77.36 (C3), 76.82 (C3), 76.37 (C3), 76.06 (C3), 75.38 (C4), 75.20 (C4), 74.81 (C4), 74.38 (C4), 73.48 (C6), 65.05 (C5), 64.09 (C5), 63.69 (C5), 62.47 (C5), 55.78 (C9), 55.00 (C9), 53.04 (C9), 32.25 (C12 or C13), 32.17 (C12 or C13), 24.19 (C12 or C13), 23.94 (C12 or C13), 23.79 (C12 or C13), 23.37 (C12 or C13). **^1^H NMR** (500 MHz, CD_3_OD) fraction B (mixture of two diastereoisomers): *δ* 6.20 (d, *J* = 5.1 Hz, 2H, H6A), 6.17 (d, *J* = 5.1 Hz, 2H, H6B), 5.80 (t, *J* = 5.2 Hz, 4H, H5A + H5B), 5.67 (d, *J* = 5.4 Hz, 2H, H4A), 5.64 (d, *J* = 5.3 Hz, 2H, H4B), 5.23 (dd, *J* = 13.5, 5.2 Hz, 2H, H9A), 4.67 (dd, *J* = 12.6, 5.3 Hz, 2H, H9B), 4.18 (d, *J* = 15.1 Hz, 2H, H8A), 4.07 (d, *J* = 14.8 Hz, 2H, H8B), 3.67 (d, *J* = 15.1 Hz, 2H, H8A), 3.42 (d, *J* = 14.8 Hz, 2H, H8B), 3–2 (m, 8H, H12A + H12B + H13A + H13B). **^13^C NMR** (125 MHz, CD_3_OD) fraction B (mixture of two diastereoisomers): *δ* 174.40 (C=O), 174.18 (C=O), 172.89 (C=O), 172.35 (C=O), 171.93 (C=O), 171.55 (C=O), 122.13 (C1), 121.91 (C1), 82.04 (C2), 81.83 (C2), 76.26 (C3), 75.38 (C3), 73.88 (C4), 73.16 (C4), 72.95 (C6), 62.88 (C5), 55.82 (C9), 53.09 (C9), 47.99 (C8), 44.48 (C8), 23.91 (C12 or C13), 23.37 (C12 or C13). **^1^H NMR** (500 MHz, CD_3_OD) fraction C, (U,*R;* U,*R*) and (D,*S;* D,*S*): *δ* 6.20 (d, *J* = 5.1 Hz, 2H, H6), 5.83 (t, *J* = 5.2 Hz, 2H, H5), 5.64 (d, *J* = 5.4 Hz, 2H, H4), 5.26 (m, 2H, H9), 4.09 (d, *J* = 14.9 Hz, H8), 3.44 (d, *J* = 14.8 Hz, H8), 2.88 and 2.33 (m, 4H, H12 + H13).**^13^C NMR** (125 MHz, CD_3_OD) fraction C, (U,*R;* U,*R*) and (D,*S;* D,*S*): *δ* 174.2 (C=O), 173.0 (C=O), 172.0 (C=O), 121.9 (C1), 81.7 (C2), 75.9 (C3), 74.0 (C4), 73.4 (C6), 63.5 (C5), 53.0 (C9), 44.5 (C8), 32.3 (C12 or C13), 24.0 (C12 or C13). **HRMS (ESI^+^):** *m/z* calcd for C_26_H_26_N_6_O_6_Re [M]^+^ 705.14658; found 705.14582.

### 4.3 Synthesis of [Re(η^6^‐C_6_H_6_)(η^6^‐lena)]TFA (2TFA)

[Re(η^6^‐C_6_H_6_)(η^6^‐naphthalene)]PF_6_ (30 mg, 0.056 mmol) was partially dissolved in 5 mL of dioxane and mixed with lenalidomide (180.0 mg, 0.70 mmol, in excess) and NMP (27 µL, 0.28 mmol, in excess) in a Schlenk tube, which was heated at 120 °C for 16 h. The solvent was removed under vacuum and the residue was purified by preparative RP‐HPLC/**G2** gradient (given in SI) and freeze‐dried to afford **2TFA** (14 mg, 58%) as a pale yellow solid. Single crystals, suitable for X‐ray diffraction analysis were obtained by vapor diffusion from Et_2_O (antisolvent) into methanol (solvent). ^1^H NMR (400 MHz, CD_3_OD) δ: 6.37 (d, *J* = 5.1 Hz, 1H, H6 minor diastereoisomer), 6.34 (d, *J* = 5.1 Hz, 1H, H6 major diastereoisomer), 6.26 (m, 2H, H5, both diastereoisomer), 6.07 (m, 2H, H4 both diastereoisomer), 5.79 (s, 6H, benzene minor diastereoisomer), 5.77 (s, 6H, benzene major diastereoisomer), 5.21 (m, 1H, H9 minor diastereoisomer), 4.69 (m, 1H, H9 major diastereoisomer), 4.19 (m, 4H, H8 both diastereoisomer), 2.95–2.10 (m, 8H, H12 + H13 both diastereoisomer). ^13^C NMR (101 MHz, CD_3_OD) *δ*: 174.56 (C=O), 171.48 (C=O), 172.08 (C=O), 123.33 (C1), 82.25 (C2), 79.25 (C3), 78.97 (benzene minor diastereoisomer), 78.79 (benzene major diastereoisomer), 76.32 (C4), 76.18 (C4), 71.94 (C6), 64.21 (C5), 55.77 (C9), 52.83 (C8), 51.67 (C8), 32.18 (C12 or C13), 23.81 (C12 or C13), 23.50 (C12 or C13). HRMS (ESI^+^) m/z calcd. For C_19_H_19_O_3_N_3_Re [M]^+^: 524.09784, found: 524.09820.

### 4.4 Synthesis of [^99m^Tc(η^6^‐lena)_2_]TFA (3TFA)

Zn (15.5 mg powder or 27 mg turnings), SDS (5 mg), lenalidomide (0.22 mmol = 57 mg), 3 N HCl (50 µL), were added into the vial. The vial was sealed and flushed with N_2_ for 1 min. Na[^99m^TcO_4_] (1 mL, 50–100 MBq) was added, the vial flushed with N_2_ for 1 min, and heated at 100 °C (μwave) for 1 h. The authenticities of the products were confirmed by coinjection with the corresponding Re analog (Figure 6), by RP‐HPLC UV/vis‐detector paired with γ‐detector using gradient G3 (given in Supporting Information). The separation of the ^99m^Tc products (3) (Figure 5, raw radio‐chromatogram) from excess ligand or residual [^99m^TcO_4_]^−^ was achieved by analytical HPLC. To get clean coinjections, all possible eight stereoisomeric pairs of [^99m^Tc(η^6^‐lena)]^+^ (3) complex were collected and then mixed with the Re homologs.

### 4.5 Recombinant CRBN Protein Binding of [Re(η^6^‐lena)_2_]^+^ (1) and [Re(η^6^‐C_6_H_6_)(lena)]^+^ (2)

The binding activity of [Re(η^6^‐lena)_2_]^+^ (1), [Re(η^6^‐C_6_H_6_)(η^6^‐lena)]^+^ (2), lenalidomide, and pomalidomide (reference) to Cereblon (CRBN) was determined using an in vitro binding assay. These binding assays were performed using the CRBN protein binding assay kit from BPS Biosciences (catalog: 79899). 5000 μM dilutions of the test compounds were prepared in assay buffer (10% DMSO concentration) and 5 µL of the dilution was added to a 50 µL reaction so that the final concentration of DMSO is 1% in all reactions. The binding reactions were conducted at room temperature for 60 min in a 50 µL mixture containing CRBN assay buffer, 10 nM of cy5‐thalidomide, cereblon, and the test compound. Fluorescence intensity was measured at an excitation of 635 nm and an emission of 682 nm using a Tecan Infinite M1000 microplate reader. CRBN binding assays were performed in duplicate at each concentration. Fluorescence intensity is converted to fluorescence polarization using the Tecan Magellan 6 software. The fluorescence polarization data were analyzed using the computer software, GraphPad Prism. The fluorescence polarization (FP_t_) in absence of the compound in each data set was defined as 100% activity. In the absence of CRBN and the compound, the value of fluorescent polarization (FP_b_) in each data set was defined as 0% activity. The percent activity in the presence of the compound was calculated according to the following equation: % activity = (FP–FP_b_)/(FP_t_−FP_b_) × 100%, where FP = the fluorescence polarization in the presence of the compound. The values of % activity versus a series of compound concentrations were then plotted using nonlinear regression analysis of Sigmoidal dose–response curve generated with the equation *Y* = *B*+(*T*−*B*)/1 + 10^((LogEC50‐X) × Hill Slope)^, where *Y* = percent activity, *B* = minimum percent activity, *T* = maximum percent activity, *X* = logarithm of compound and Hill Slope = slope factor or Hill coefficient. The IC_50_ value was determined by the concentration causing a half‐maximal percent activity. The IC_50_ values of the compounds against CRBN are summarized in Figure 8, Table 1 and in (Tables S3–S7). Approximate value is assigned for the IC_50_ when only one plateau for curve fit is available.

### 4.6 Animal Studies

All procedures were performed in accordance with the ARRIVE guidelines and the guidelines of the Canadian Council on Animal Care and were approved by the McMaster University Animal Research Ethics Board. BALB/c mice (7–8 weeks old; Charles River Laboratories, Raleigh, NC) were used for biodistribution studies. Animals were housed under sterile conditions at 20 °C with a 12 h light/dark cycle and had ad libitum access to autoclaved food and water.

### 4.7 Biodistribution Studies of [^99m^Tc(η^6^‐lena)_2_]^+^ (3) in BALB/c Mice

[^99m^Tc(η^6^‐lena)_2_]^+^ (3) was radiolabeled following standard procedures. The radioactive product was purified using preparative‐HPLC to remove the excess of free ligand and reformulated in 10% ethanol in saline. In vivo studies were performed in Balb/c mice (7–8 weeks) to get information in biodistribution and pharmacokinetics of [^99m^Tc(η^6^‐lena)_2_]^+^ (Figure 7). Mice were IV injected with 100 µL, 370 kBq (10 µCi) of 3700 kBq/mL (100 µCi/mL) of [^99m^Tc(η^6^‐lena)_2_]^+^. At specific time points post‐radioactive injection (*t* = 30, 90 min), mice (*n* = 3) were euthanized by cardiac puncture under isoflurane anesthesia followed by cervical dislocation, and blood and select tissues were collected. Tissues were weighed and the radioactivity in the samples was measured using an automated Gamma Counter (PerkinElmer Waltham, MA). The percentage of injected dose per gram of tissue (%ID/g) (Figure 7a) and per organ (%ID/o) (Figure 7b) was calculated to characterize the in vivo distribution of the radiolabeled compound. Data arw presented as average ± SEM.

## Funding

This study was supported by Universität Zürich.

## Conflicts of Interest

The authors declare no conflicts of interest.

## Supporting information

The additional references within the Supporting Information are added [27, 28, 29, 30, 31, 32].

## Data Availability

The data that support the findings of this study are available from the corresponding author upon reasonable request.
