# Supplementary material for: Chelator Free Radiolabeling and Preclinical Evaluation of η6‐Arene Sandwich Complexes of Lenalidomide With Rhenium and Technetium‐99m
Source: Chembiochem. 2026 Aug 21;27(16):e70503. doi: 10.1002/cbic.70503 (PMC13495133; doi:10.1002/cbic.70503)
Supplement: Supplementary file 1 [file CBIC-27-e70503-s001.pdf]

Supplementary Information

Chelator Free Radiolabeling and Preclinical Evaluation of η^6-Arene Sandwich Complexes of Lenalidomide with Rhenium and Technetium-99m

Qaisar Nadeem*[1,2], Federica Battistin[3], Daniel Hernández Valdés[4], Afaf Genady[4], Luis Rafael Silva Mendez[4], Saman Sadeghi[4], Olivier Blacque[2] and Roger Alberto[2]

[1]International Center for Interdisciplinary Research in Sciences, The University of Lahore, Lahore, Pakistan

[2]Department of Chemistry, University of Zurich, Zurich, Switzerland

[3]Dipartimento di Scienze Chimiche e Farmaceutiche, Università di Trieste, Trieste, Italy

[4]Department of Chemistry and Chemical Biology, McMaster University, Hamilton, Ontario, Canada

Qaisar Nadeem (nadeem_awan111@yahoo.com)

Table of Contents

Material and methods......2

Stereochemical assignments for all eight diastereomeric pairs of $[Re(\eta^6\text{-lena})_2]^+$......5

NMR spectra......6

X-ray crystallography......12

***In vitro* cellular binding of $[Re(\eta^6\text{-lena})_2]^+$ and $[Re(\eta^6\text{-}C_6H_6)(\eta^6\text{-lena})]^+$**......14

***In vivo* biodistribution studies of $[^{99m}Tc(\eta^6\text{-lena})_2]^+$ in vivo**......16

References......18

Materials and methods: Unless otherwise stated, all chemicals were of reagent grade or higher, obtained from commercial sources and used without further purification. Solvents for reactions were of p.a. grade or distilled prior to their use; H_2O was bi-distilled. Deuterated NMR-solvents were purchased from *Armar Chemicals* or *Cambridge Isotope Laboratories, Inc.* (UK). Reactions were carried out using standard *Schlenk* techniques in oven-dried (150 °C) glass equipment and monitored for completion by analyzing a small sample (after suitable workup) by UPLC or UPLC-ESI-MS. Evaporation of the solvents *in vacuo* was done with the rotary evaporator. Microwave assisted reactions were carried out in a *Biotage Initiator* microwave. Lenalidomide was purchased from Sigma Aldrich**.**

Radiochemistry: ^{99m}Tc is a radioactive isotope. All operations dealing with ^{99m}Tc must be carried out in a licensed and appropriately equipped laboratory, including state of the art radiation protection measures.

^{1}H-and^{13}C-NMR spectra: ^{1}HNMR, ^{13}CNMR and DEPT-135-^{13}C-NMR spectra were recorded on a BrukerDRX 500 MHz or BrukerDRX 400 MHz spectrometers.^{1}H and ^{13}C chemical shifts were referenced with the residual solvent resonance relative to TMS.

HR-ESI-MS: *QExactive* (*Thermo Fisher Scientific*, Bremen, Germany) equipped with a heated ESI source connected to a *Dionex Ultimate 3000* UPLC system. Samples dissolved in MeOH, MeOH/CH_2Cl_2 3:1, MeOH/H_2O 1:1, DMSO/H_2O 1:10, or H_2O at ca. 50 µg mL^{-1}; injection of 1 µL on-flow with an XRS auto-sampler (*CTC*, Zwingen, Switzerland)(mobile phase: MeOH + 0.1% HCOOH or CH_3CN/H_2O (2:8) + 0.1% HCOOH; flow rate 120 µL mL^{-1}); ion source parameters: spray voltage 3.0 kV, capillary temperature 280 °C, sheath gas 30 L min^{-1}, s-lens RF level 55.0; aux gas temperature 250 °C; full scan MS in alternating (+)/(−)-ESI mode; mass ranges 80–1’200, 133–2’000, or 200–3’000 amu; resolution (full width half-maximum) 70’000; automatic gain control(AGC) target 3.00 10^{6}; maximum allowed ion transfer time(IT) 30ms; mass calibration < 2 ppm accuracy for *m/z* 130.06619–1621.96509 in (+)-ESI with *Pierce*® ESI calibration solutions (*Thermo Fisher Scientific*, Rockford, USA); lock masses: ubiquitous erucamide (*m/z*338.34174,(+)-ESI).

UPLC-ESI-MS: *Waters Acquity* UPLC System coupled to a *Bruker Daltonics HCT™*ESI-MS, using an *Acquity UPLC BEH* C18 1.7 µm (2.1 x 50 mm) column. UPLC solvents were formic acid (0.1% in millipore water) (solvent A) and acetonitrile UPLC grade (solvent B). Applied UPLC gradient: 0–0.5min: 95% A, 5% B; 0.5–4.0 min: linear gradient from 95% A, 5% B to 0% A, 100% B; 4.0–5.0 min: 0% A, 100% B. The flow rate was 0.6 mL min^{-1}. Detection was performed at 250 and 480 nm (DAD).

Analytical UPLC: *VWR HITACHI Chromaster Ultra*system, using an *Acquity UPLC BEH*C18 1.7µm(2.1x50mm) column. UPLC solvents were trifluoroacetic acid (0.1% in millipore water) (solvent A) and acetonitrile UPLC grade (solvent B). Applied UPLC gradient: 0–0.5 min: 95% A, 5% B; 0.5–4 min: linear gradient from 95% A, 5% B to 0% A, 100% B; 4–5 min: 0% A, 100% B. The flow rate was 0.5 mL min^{-1}. Detection was performed at 250 and 480 nm (DAD).

Preparative RP-HPLC Shimadzu eco LC-20AP system, using a Dr. Maisch Reprosil C18 100-7 (40 x250mm) column. HPLC solvents were trifluoroacetic acid (0.1% in Millipore water) (solvent A) and HPLC grade acetonitrile (solvent B), with flow rate 40mL/min.

Gradient for [Re(η^6-lena)$_2$]$^+$ (**1**) (**G1**): detection at 300 nm, 0-10 min: 95% A (5% B); 10-70 min: linear gradient from 95% A (5% B) to 85% A (15% B); 70-80 min: 85% A (15% B); 80-110 min: 85% A (15% B) to 80% A (20% B); 110-120 min: 80% A (20% B); 120-140 min: 80% A (20% B) to 50% A (50% B); 140-150 min: 50% A (50% B) to 0% A (100% B).
Gradient for [Re(η^6-C_6H_6)(η^6-lena)]$^+$ (**2**) (**G2**): detection at 283 nm, 0-5 min: 95% A (5% B); 5-40min: linear gradient from 95% A (5% B) to 60% A (40% B); 40-45 min: 60% A (40% B) to 0% A (100% B); 45-55 min: 100% B.

X-ray diffraction: Single crystal X-ray diffraction data were collected at 160.0(1) K on a Rigaku OD Synergy/Pilatus diffractometer for the structures of [Re(η^6-lena)$_2$]$^+$ (1) and on a Rigaku OD Supernova/Atlas diffractometer for the structures of [Re(η^6-C_6H_6)(η^6-lena)]$^+$ (2), using the Cu Kα radiation (λ= 1.54184 Å) from a dual wavelength X-ray source and an Oxford Instruments Cryojet XL cooler. The selected suitable single crystals were mounted using polybutene oil on a flexible loop fixed on a goniometer head and immediately transferred to the diffractometer. Pre-experiment, data collection, data reduction and analytical absorption correction[27] were performed with the program suite *CrysAlisPro*.[28] Using *Olex2*,[29] the structures were solved with the *SHELXT*[30] small molecule structure solution program and refined with the *SHELXL* program package[31] by full-matrix least-squares minimization on F^2. *PLATON*[32] was used to validate the results of the X-ray analyses. CCDC2538046-2538049 contain the supplementary crystallographic data for this paper. The data can be obtained free of charge from The Cambridge Crystallographic Data Centre via www.ccdc.cam.ac.uk/structures.
In the crystal structure of [Re(η^6-lena)$_2$]$^+$ (1) in fraction B, enantiomeric pair (U,S; U,R)/(D,R; D,S), the counter-ion is severely disordered and was modelled as a superposition of ReO_4^- and CF_3COO^- anions. The refined site-occupancy factors are 0.144(4) and 0.856(4), respectively. Geometrical restraints (DANG, DFIX, SADI) were applied to maintain chemically reasonable bond distances and angles in the disordered components, while the displacement parameters of the corresponding atoms were restrained (SIMU) to ensure physically reasonable anisotropic behaviour. Solvent molecules of water co-crystallize with the main species. The site occupancy was refined to 0.74(4). In the main species, the H atoms attached to the N atoms were located in a difference Fourier map. Their positions were refined using a riding model with N—H = 0.86–0.88 Å, while their isotropic displacement parameters were freely refined. In the crystal structure of [Re(η^6-lena)$_2$]$^+$ (1) of fraction C, enantiomeric pair (U,R; U,R)/(D,S; D,S), the CF_3COO^-counter-ion is disordered over two sets of positions with refined site-occupancy factors of 0.384(8) and 0.616(4). Geometrical restraints (DANG, DFIX, FLAT) were applied to maintain chemically reasonable bond distances and angles in the disordered components, while the displacement parameters of the corresponding atoms were restrained (ISOR, SIMU) to ensure physically reasonable anisotropic behaviour. The H atoms attached to the N atoms were located in a difference Fourier map. Their positions were refined using a riding model with N—H = 0.88–0.91 Å, while their isotropic displacement parameters were freely refined. In the crystal structure of [Re(η^6-C_6H_6)(η^6-lena)]$^+$ (2), enantiomeric pair (U,R)/D,S), the CF_3COO^- counter-ion is disordered over two sets of positions with refined site-occupancy factors of 0.384(7) and 0.616(7). Geometrical restraints (DANG, DFIX) were applied to maintain

chemically reasonable bond distances and angles in the disordered components, while the displacement parameters of the corresponding atoms were restrained (ISOR, SIMU) to ensure physically reasonable anisotropic behaviour. The H atoms attached to the N atoms were located in a difference Fourier map. Their positions were refined using a riding model with N—H = 0.81–0.82 Å, while their isotropic displacement parameters were freely refined. All remaining H atoms were placed geometrically and refined using a riding model, with C—H = 0.95 Å (aromatic C—H), 0.99 Å (methylene C—H) and 1.00 Å (methine C—H), with U_{iso}(H) = 1.2U_{eq}(C). In the crystal structure of $[Re(\eta^6-C_6H_6)(\eta^6\text{-lena})]^+$ (2), enantiomeric pair (U,S)/(D,R), the asymmetric unit consits of two independent rhenium cations and two CF_3COO^- counter-ions. Both counter-ions are disordered over two sets of positions with refined site-occupancy factors of 0.340(7) and 0.660(7). Geometrical restraints (DANG, DFIX, FLAT, SADI) were applied to maintain chemically reasonable bond distances and angles in the disordered components, while the displacement parameters of the corresponding atoms were restrained (ISOR, SIMU) to ensure physically reasonable anisotropic behaviour. The H atoms attached to the N atoms were located in a difference Fourier map. Their positions were refined using a riding model with N—H = 0.88–0.94 Å, while their isotropic displacement parameters were freely refined. The structure has been refined in the non-centrosymmetric space group $P2_1$ as a 2-component inversion twin. Additional symmetries are found by PLATON (centre of symmetry 100% and c plane 88%) but all reflection statistics, Wilson plot and cumulative intensity distribution speak for a non-centrosymmetric space group. Solution and refinement in $P2_1/c$ have been tried and have resulted to higher R_1 and wR_2 in comparison with $P2_1$. In the four crystal structures, all remaining H atoms were placed geometrically and refined using a riding model, with C—H = 0.95 Å (aromatic C—H), 0.99 Å (methylene C—H) and 1.00 Å (methine C—H), with U_{iso}(H) = 1.2U_{eq}(C).

Radioactive materials: Na[^{99m}TcO$_4$] in 0.9% saline was eluted from a ^{99}Mo/^{99m}Tc *Ultratechnekow® FM* generator purchased from *b.e. imaging AG* (Switzerland).

HPLC analyses of ^{99m}Tc complex: *Merck Hitachi Chromaster 5160* pump coupled to a *Merck Hitachi Chromaster 5430* diode array detector and a radiodetector. UV-vis detection was performed at 250 nm. The detection of radioactive ^{99m}Tc complexes was performed with a *Berthold FlowStar LB 514* radiodetector equipped with a *BGO-X* cell. Separations were achieved on a *Macherey-Nagel NUCLEOSIL®*C18 5 µm, 100 Å (250 × 3 mm) column. HPLC solvents were trifluoroacetic acid (0.1% in Millipore water) (solvent A) and HPLC grade acetonitrile (solvent B). Applied HPLC gradient for co-injection of [^{99m}Tc(lenalidomide)$_2$]$^+$ (**G3**): 0-3 min: 95% A (5% B); 3-3.1 min: 95% A (5% B) to 75% A (25% B); 3.1-9 min: 75% A (25% B); 9-9.1 min: 75% A (25% B) to 66% A (34% B); 9.1-20 min: 66% A (34% B) to 0% A (100% B); 20-25 min: 0% A (100% B); 25-25.1 min: 0% A (100% B) to 95% A (5%B); 25.1-30 min: 95% A (5% B).

Stereochemical assignments for all eight diastereomeric pairs of Re(η^6-lenalidomide)$_2$]$^+$

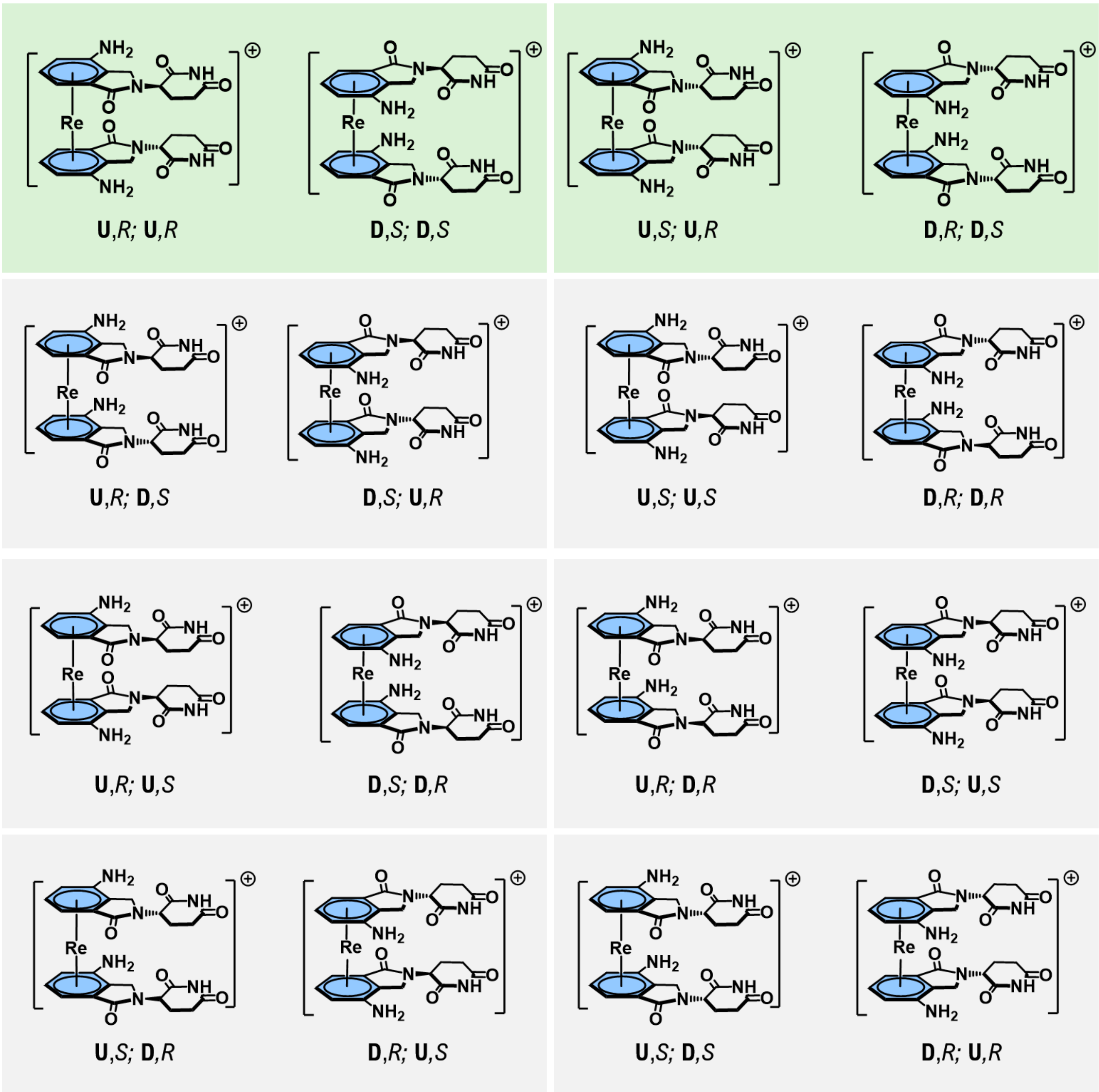

Figure S1. Eight enantiomeric pairs of [Re(η^6-lena)$_2$]$^+$ arising from the combination of two stereogenic centers (*R*/*S* on each lenalidomide) and two planar chiral elements (**U**/**D** orientation of each arene), affording 16 distinct stereochemical configurations total. Green backgrounds highlight the two diastereomeric pairs for which single-crystal X-ray structures were obtained.

NMR Spectra

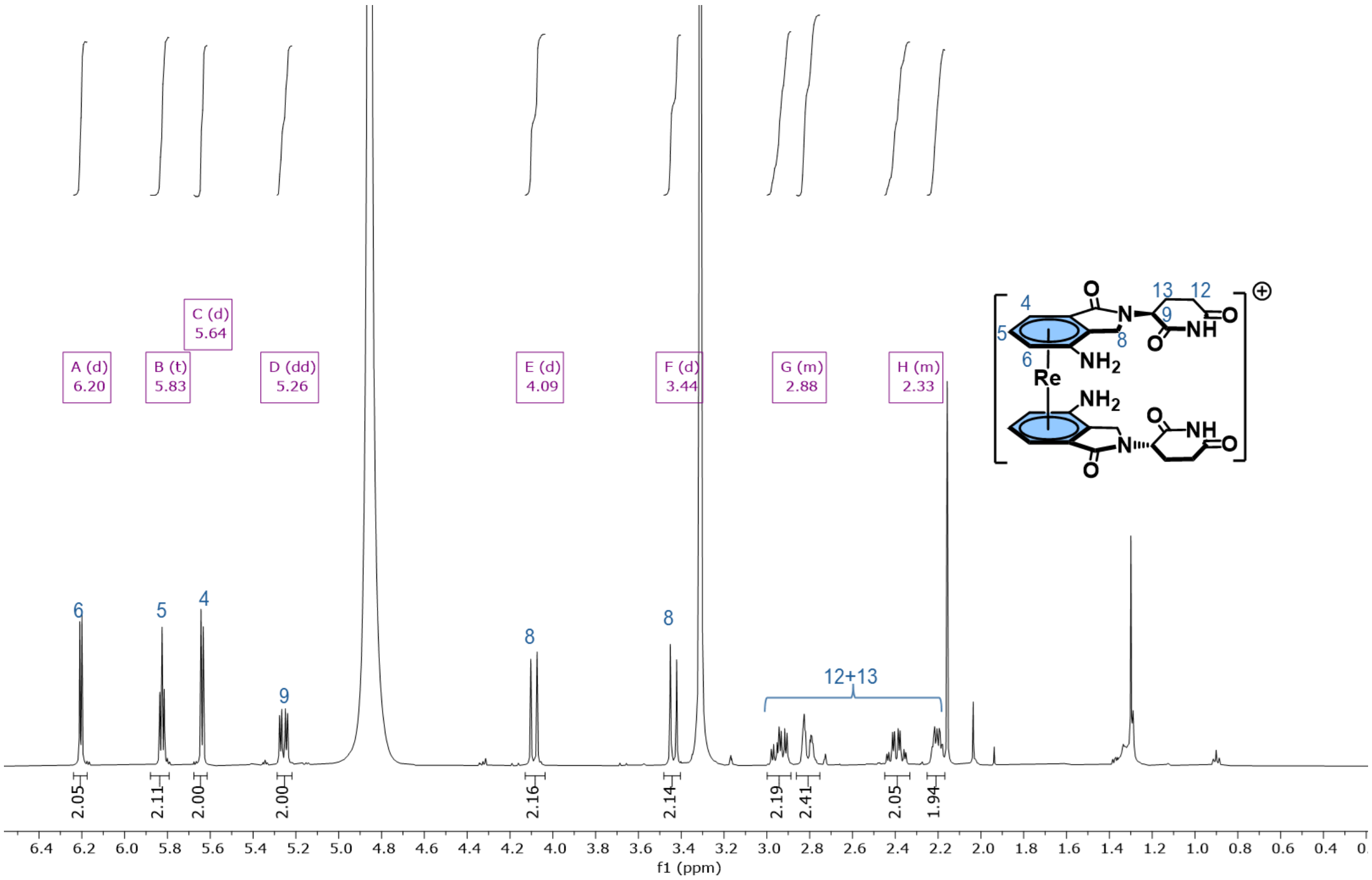

Figure S2. ^{1}H NMR spectrum in CD_3OD of Fraction C, [η^6-Re(lenalidomide)$_2$]$^+$ (**1**), enantiomeric pair (**U**,*R;* **U**,*R*) and (**D**,*S;* **D**,*S*).

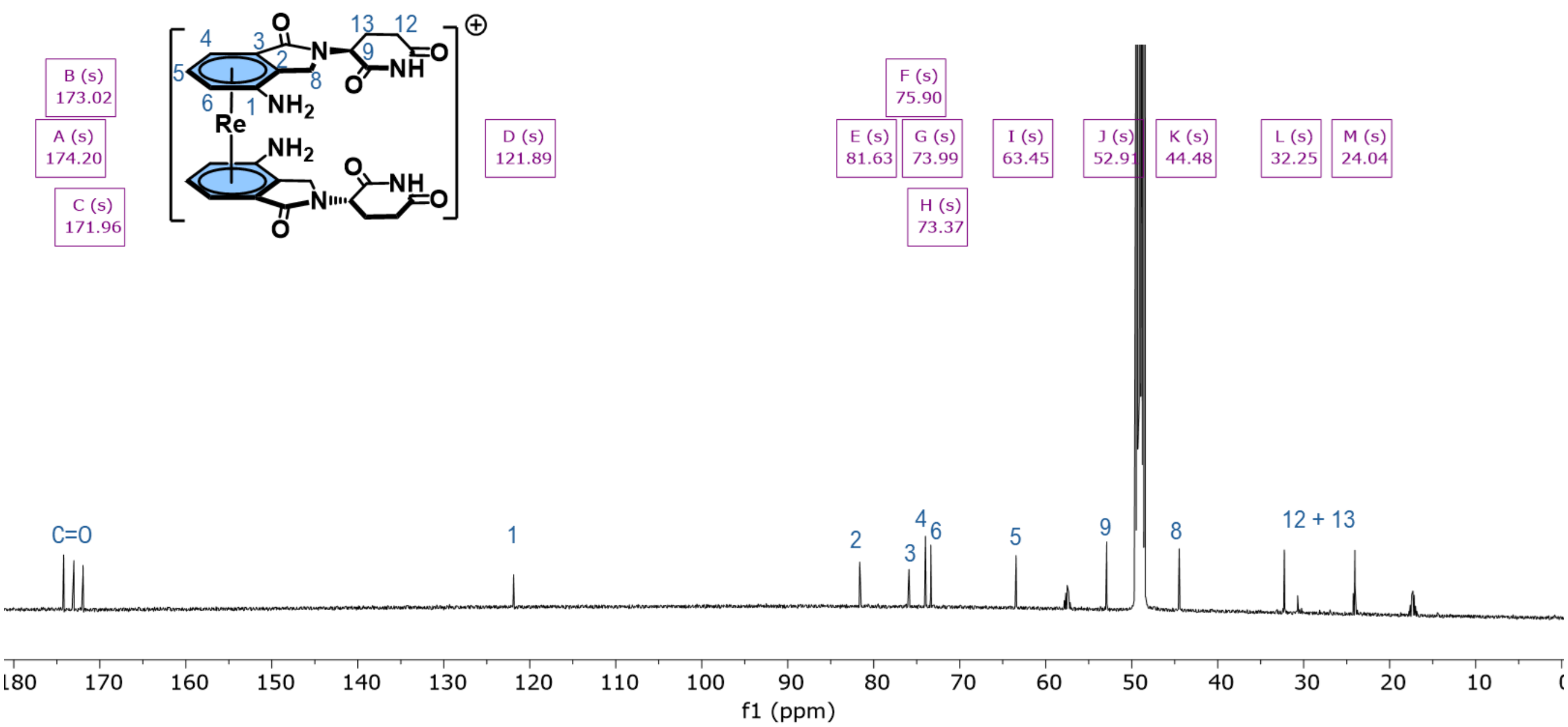

Figure S3. ^{13}C NMR spectrum CD_3OD of Fraction C, [η^6-Re(lenalidomide)$_2$]$^+$ (**1**), enantiomeric pair (**U**,*R;* **U**,*R*) and (**D**,*S;* **D**,*S*).

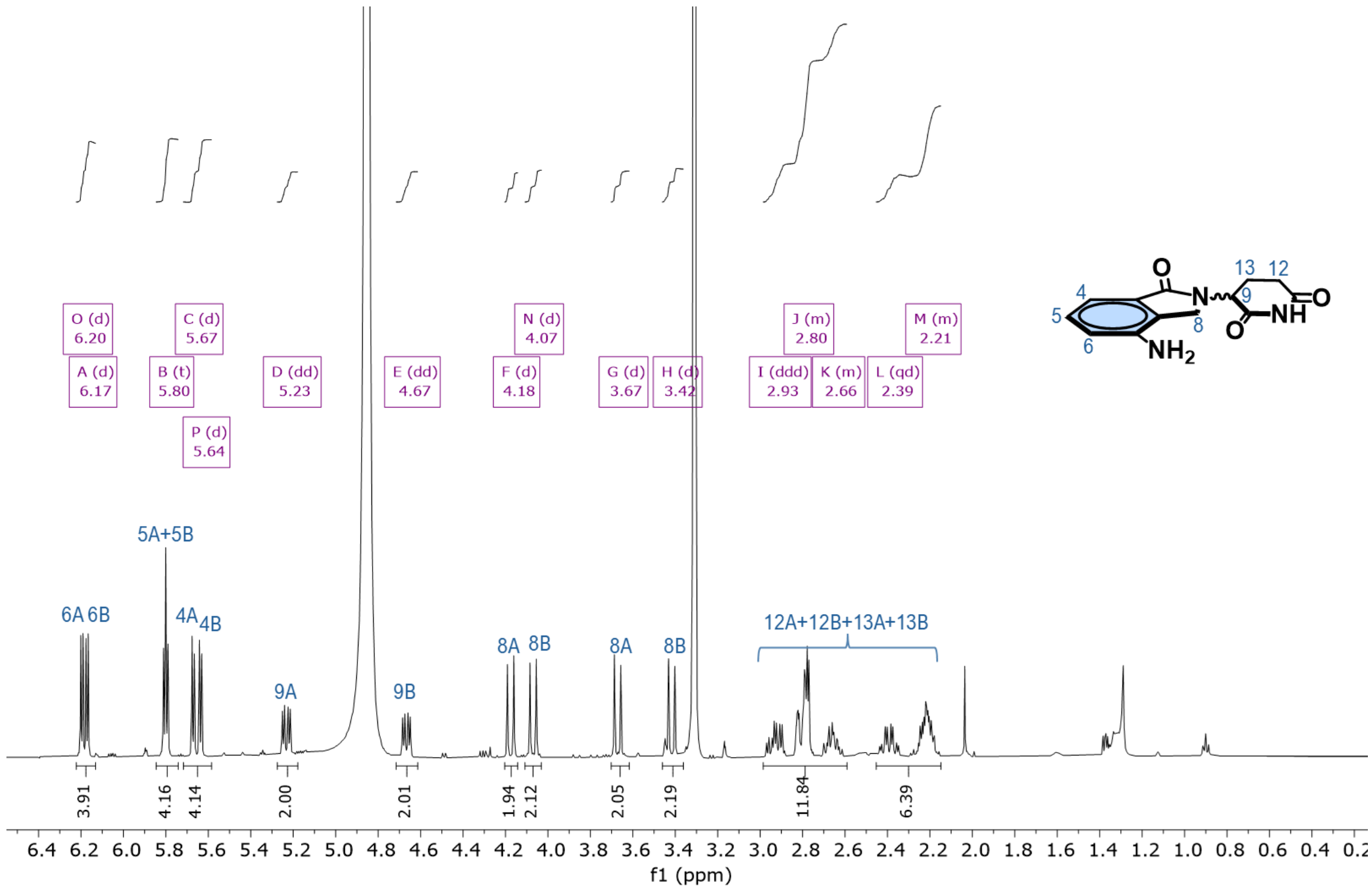

Figure S4. ^{1}H NMR spectrum in CD_3OD of Fraction B, $[\eta^6\text{-Re(lenalidomide)}_2]^+$ (**1**), mixture of two diastereoisomers. Labelling scheme on lenalidomine.

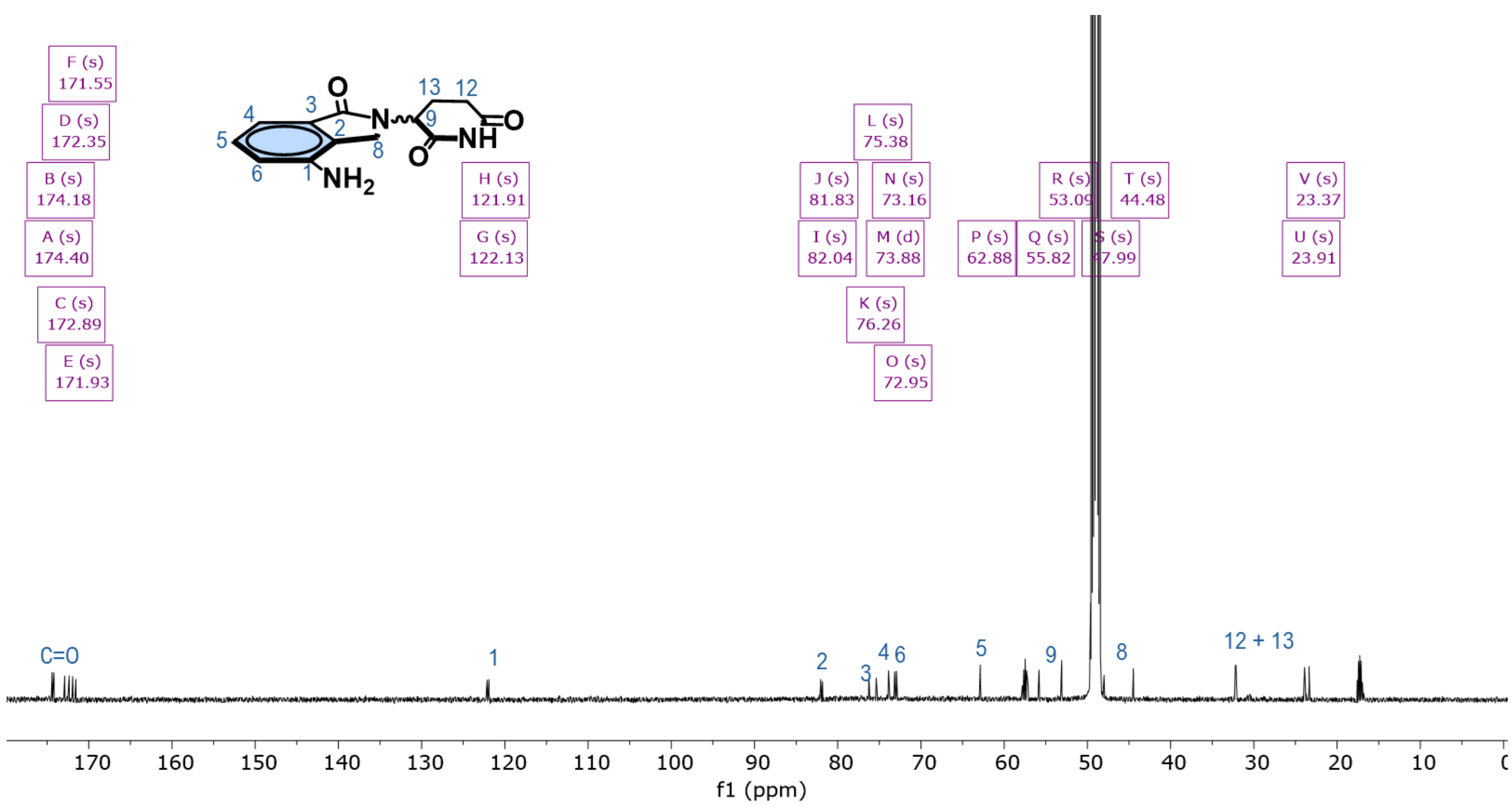

Figure S5. ^{13}C NMR spectrum in CD_3OD of Fraction B, $[\eta^6\text{-Re(lenalidomide)}_2]^+$ (**1**), mixture of two diastereoisomers. Labelling scheme on lenalidomine.

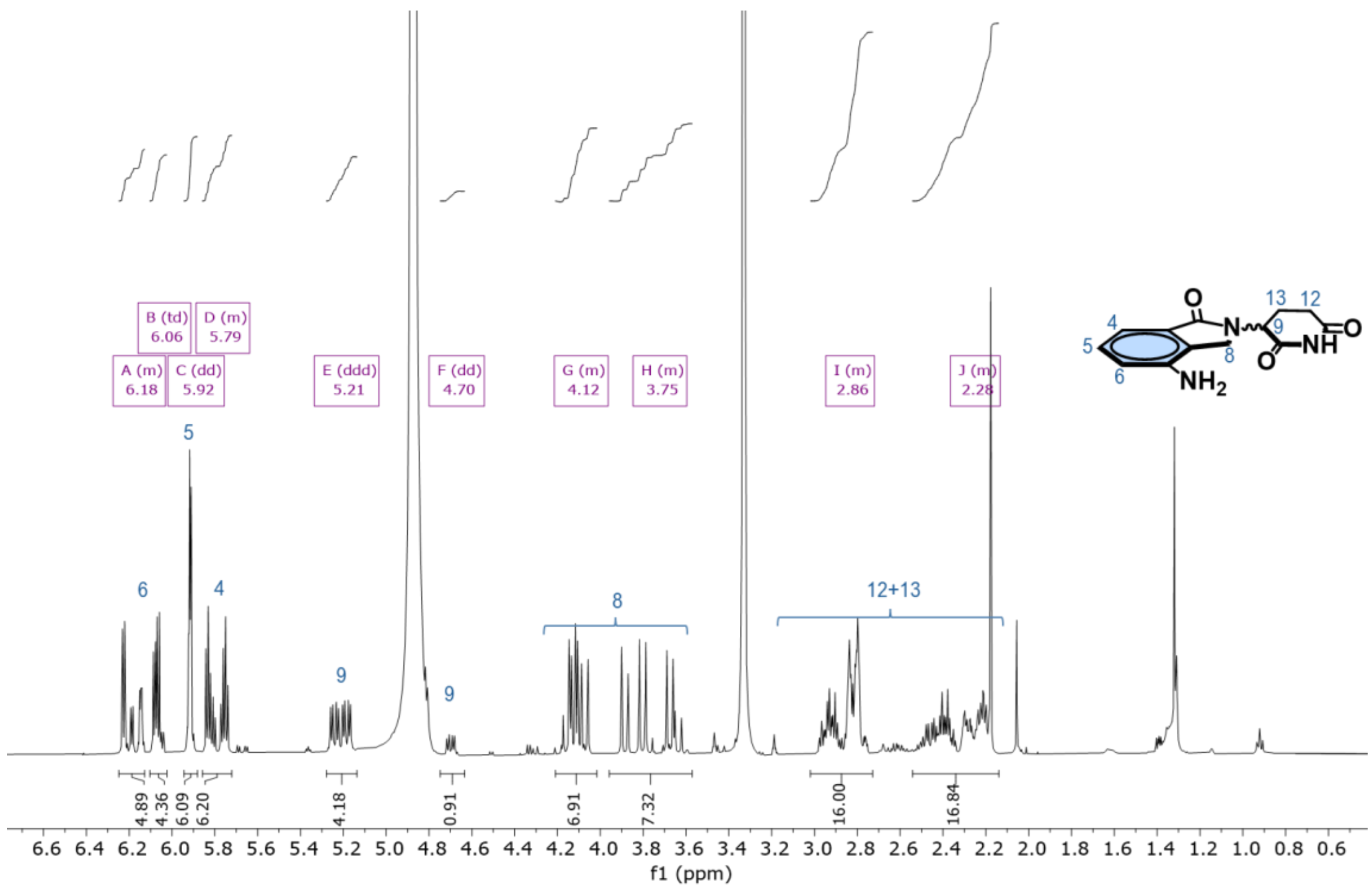

Figure S6. ^{1}H NMR spectrum in CD_3OD of Fraction A, [η^6-Re(lenalidomide)$_2$]$^+$ (**1**), mixture of at least four diastereoisomers. Labelling scheme on lenalidomine.

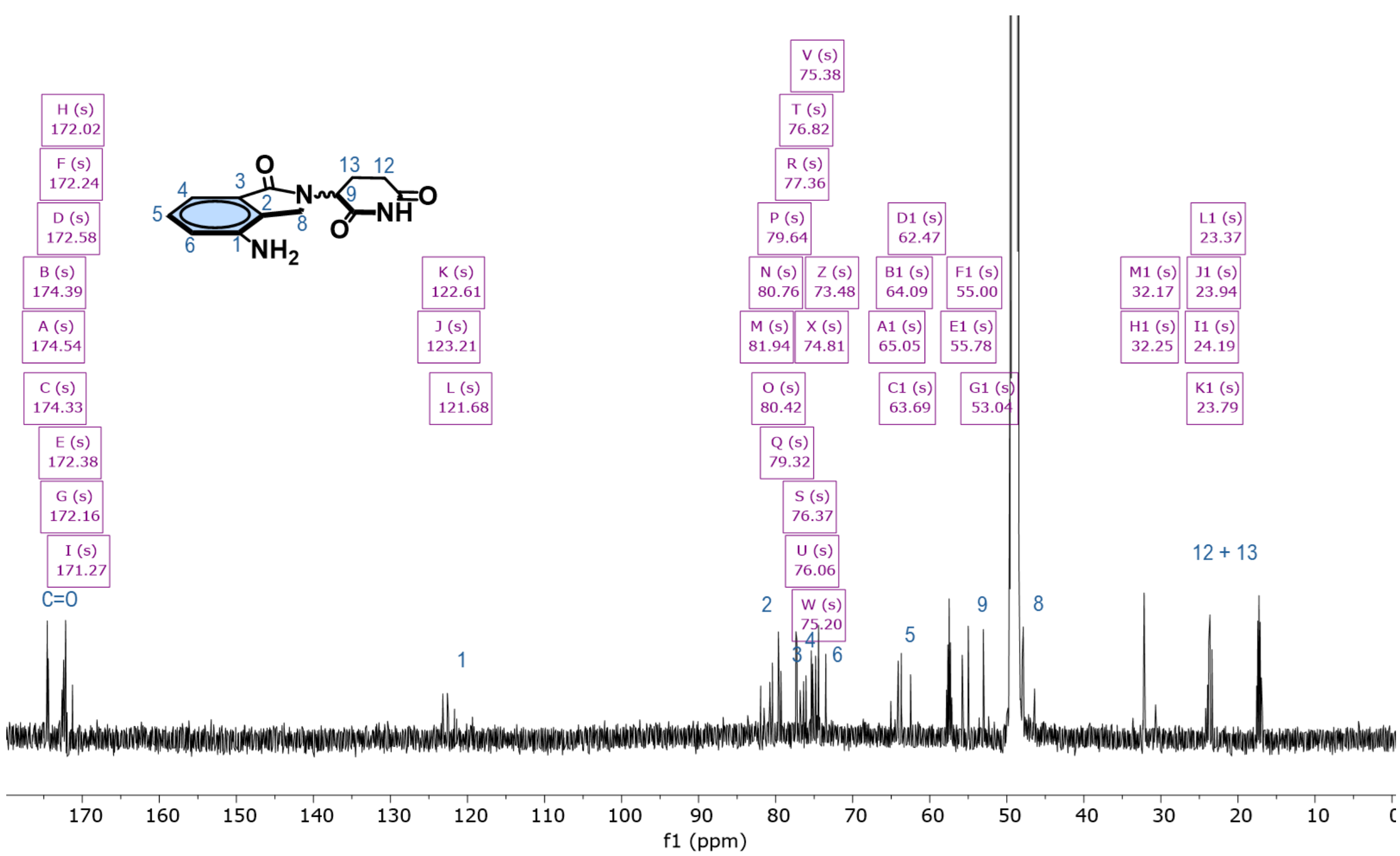

Figure S7. ^{13}C NMR spectrum in CD_3OD of Fraction A, [η^6-Re(lenalidomide)$_2$]$^+$ (**1**), mixture of at least four diastereoisomers. Labelling scheme on lenalidomine.

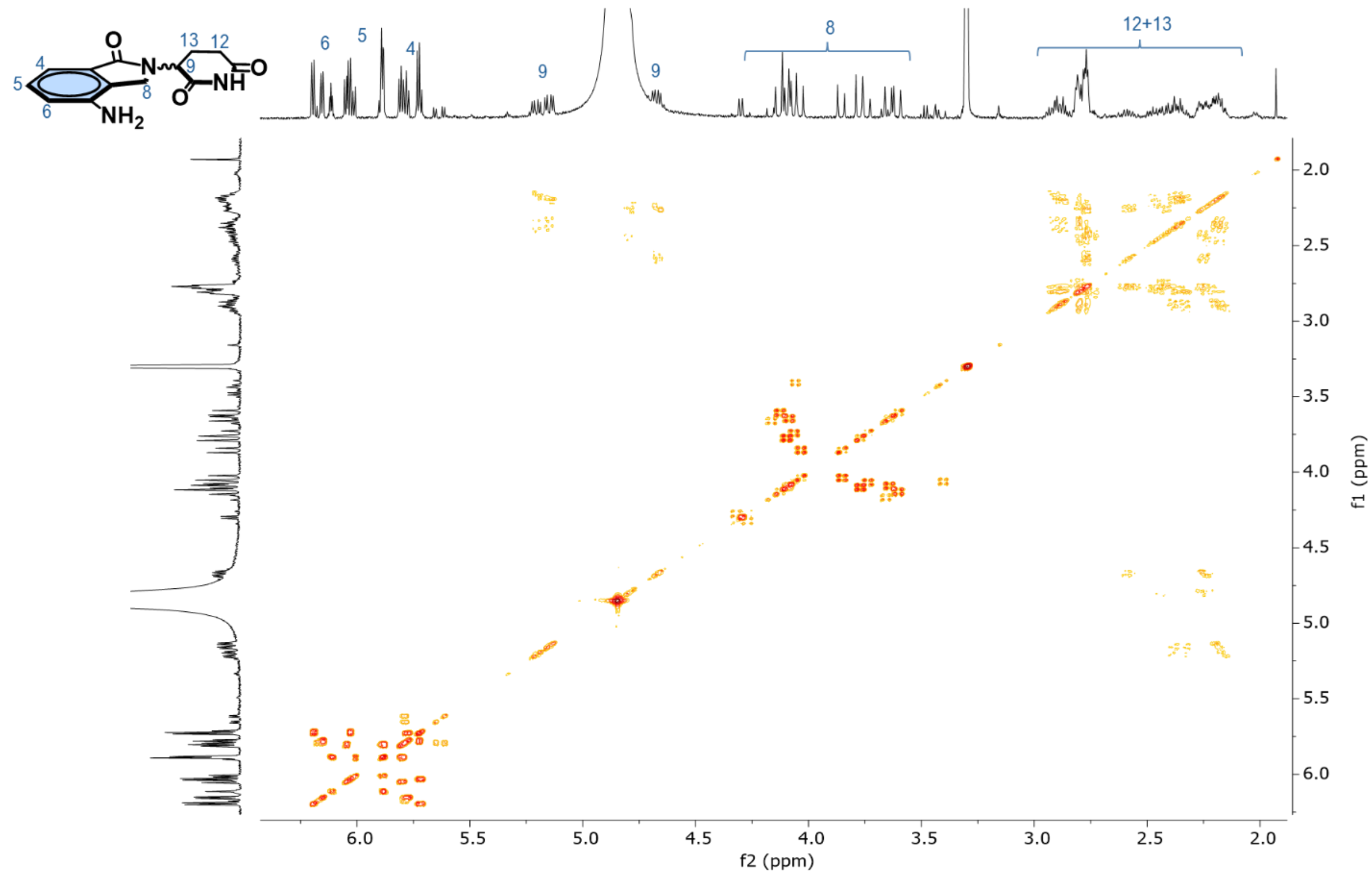

Figure S8. ^{1}H-^{1}H COSY NMR spectrum in CD_3OD of Fraction A, [η^6-Re(lenalidomide)$_2$]$^+$ (**1**), mixture of at least four diastereoisomers. Labelling scheme on lenalidomine.

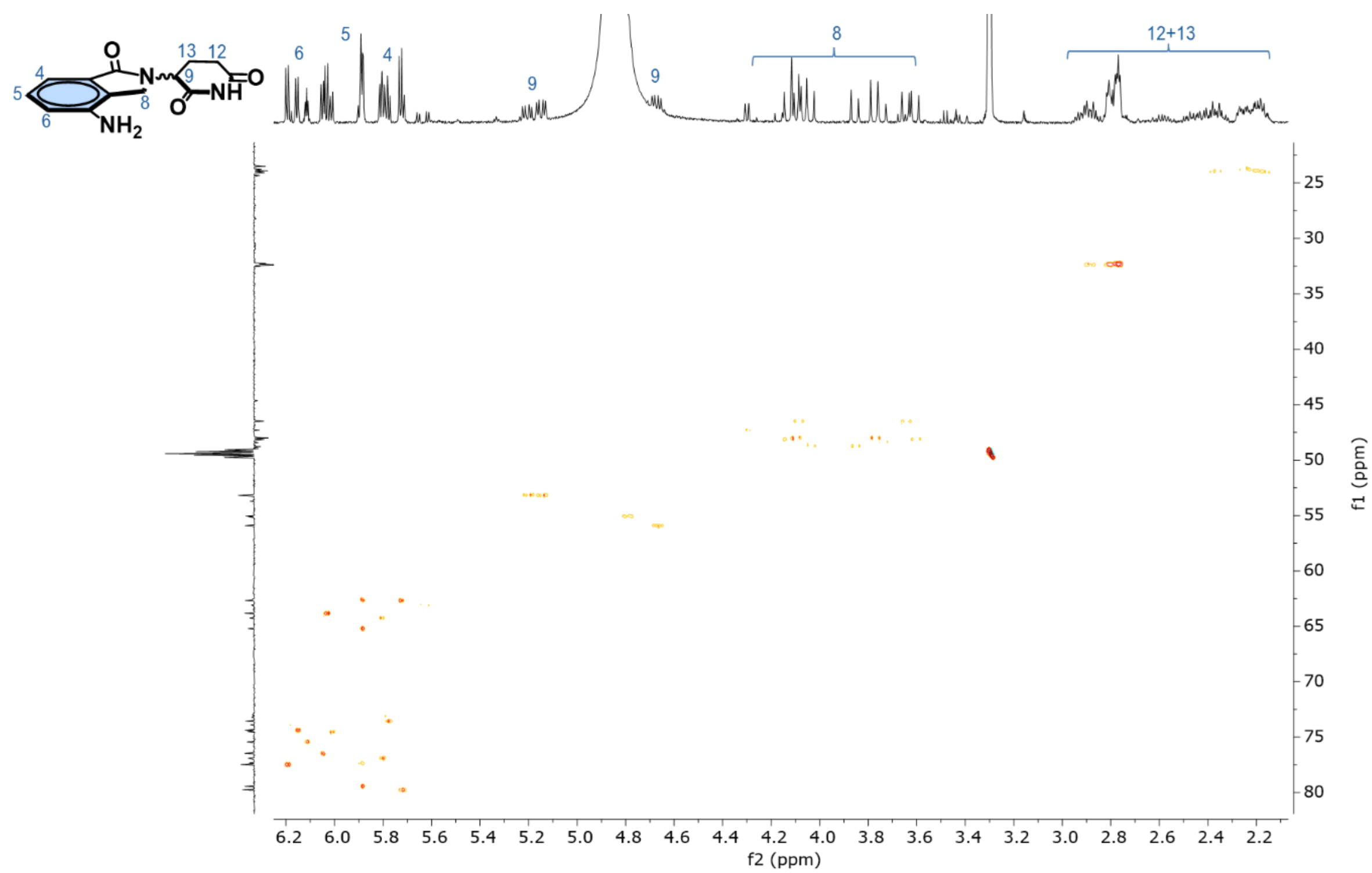

Figure S9. ^{1}H-^{13}C HSQC NMR spectrum in CD_3OD of Fraction A, [η^6-Re(lenalidomide)$_2$]$^+$ (**1**), mixture of at least four diastereoisomers. Labelling scheme on lenalidomine.

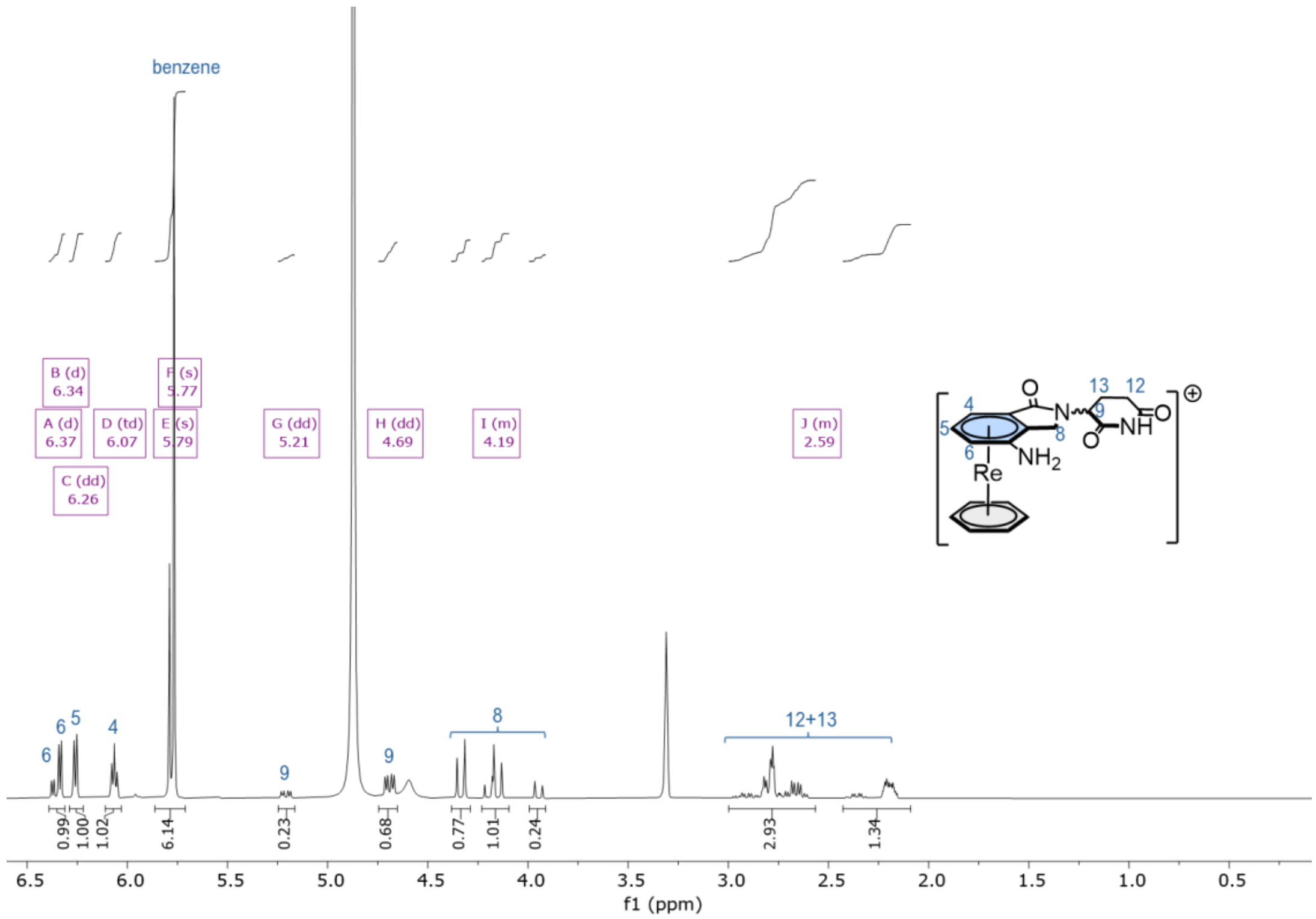

Figure S10. ^{1}H NMR spectrum in CD_3OD of [Re(η⁶-benzene)(η⁶-lenalidomide)]⁺ (**2**), both diastereoisomers.

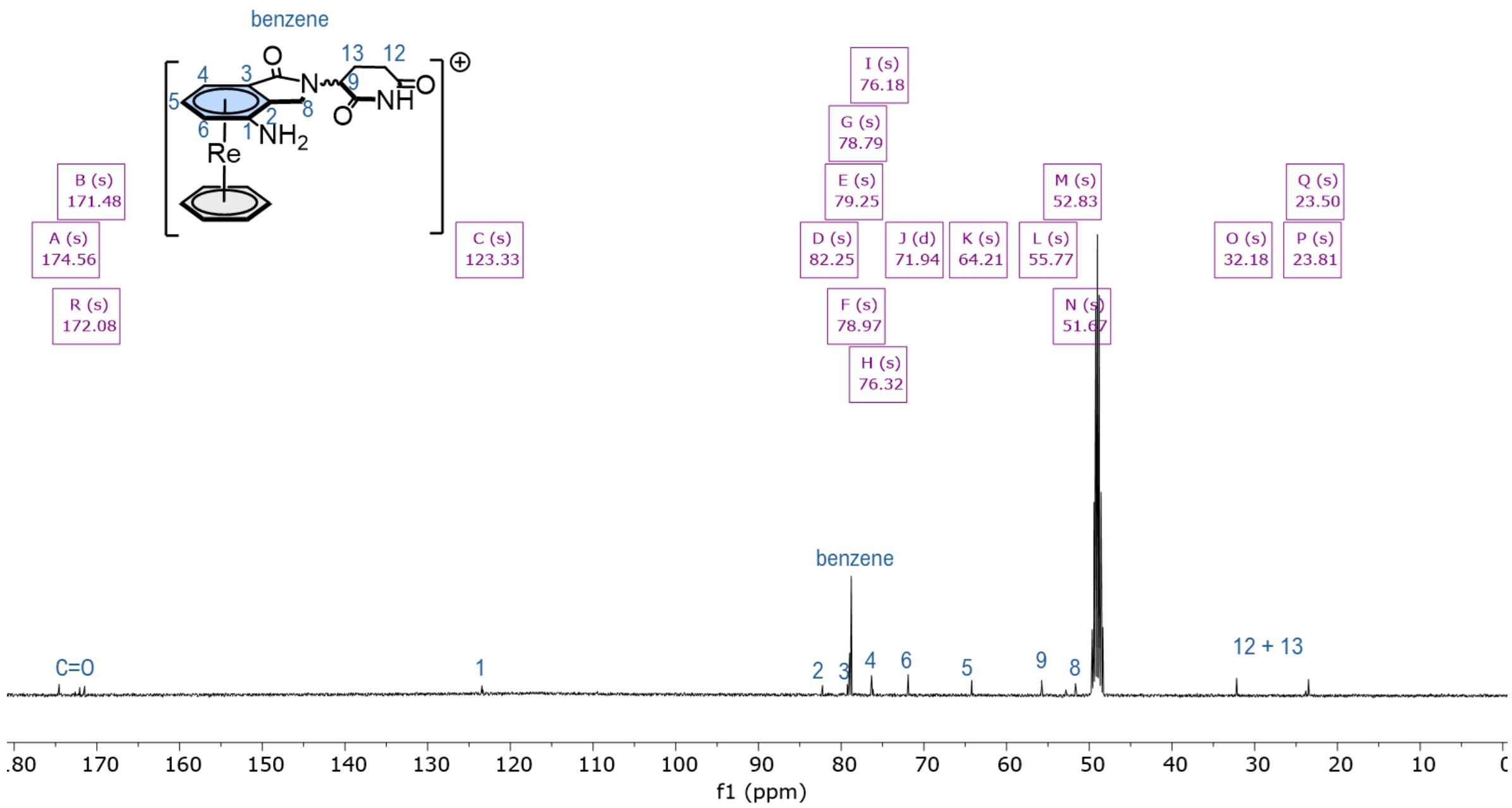

Figure S11. ^{13}C NMR spectrum in CD_3OD of [Re(η⁶-benzene)(η⁶-lenalidomide)]⁺ (**2**), both diastereoisomers.

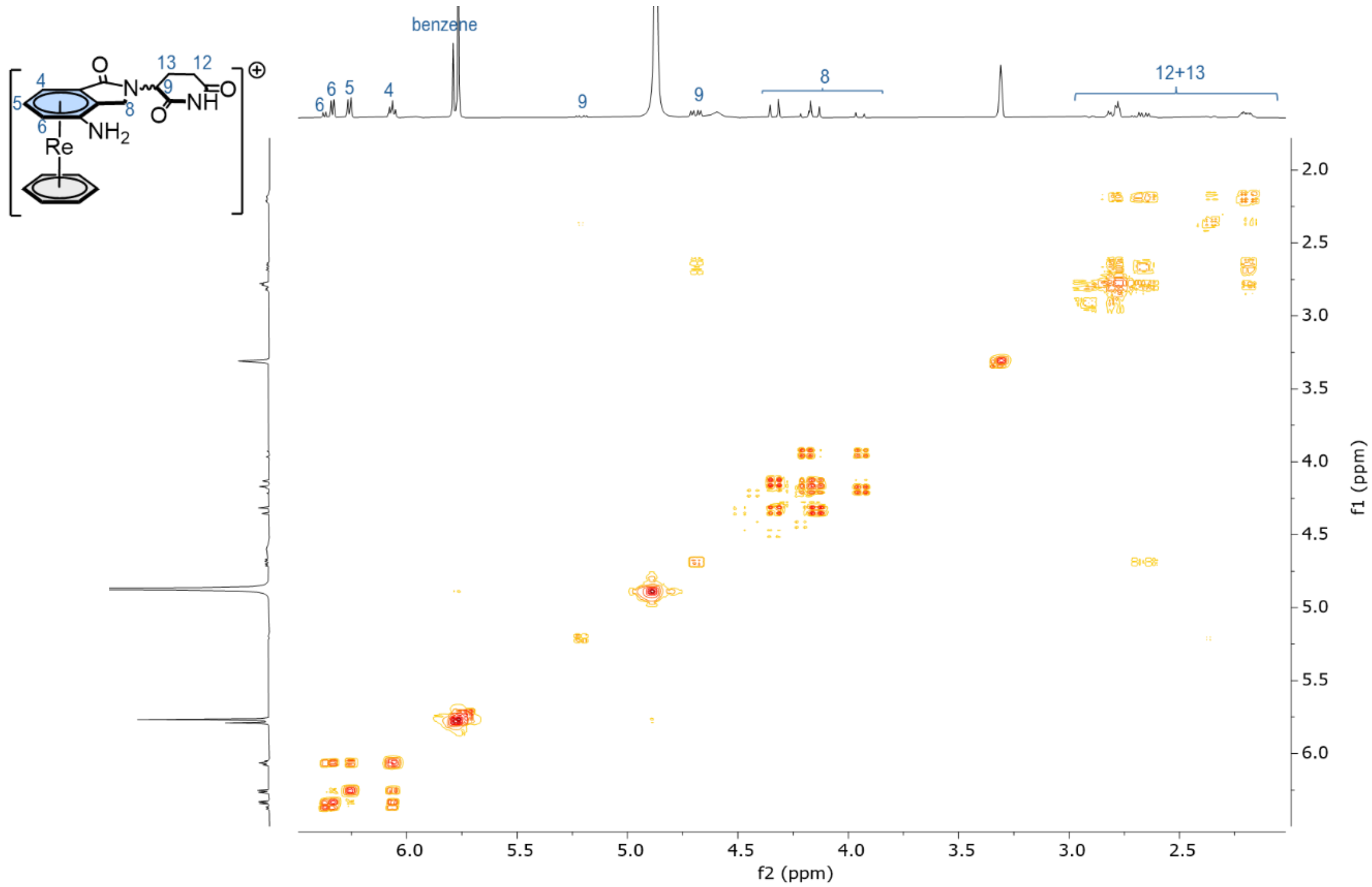

Figure S12. ^{1}H-^{1}H COSY NMR spectrum in CD_3OD of [Re(η⁶-benzene)(η⁶-lenalidomide)]$^{+}$ (**2**), both diastereoisomers.

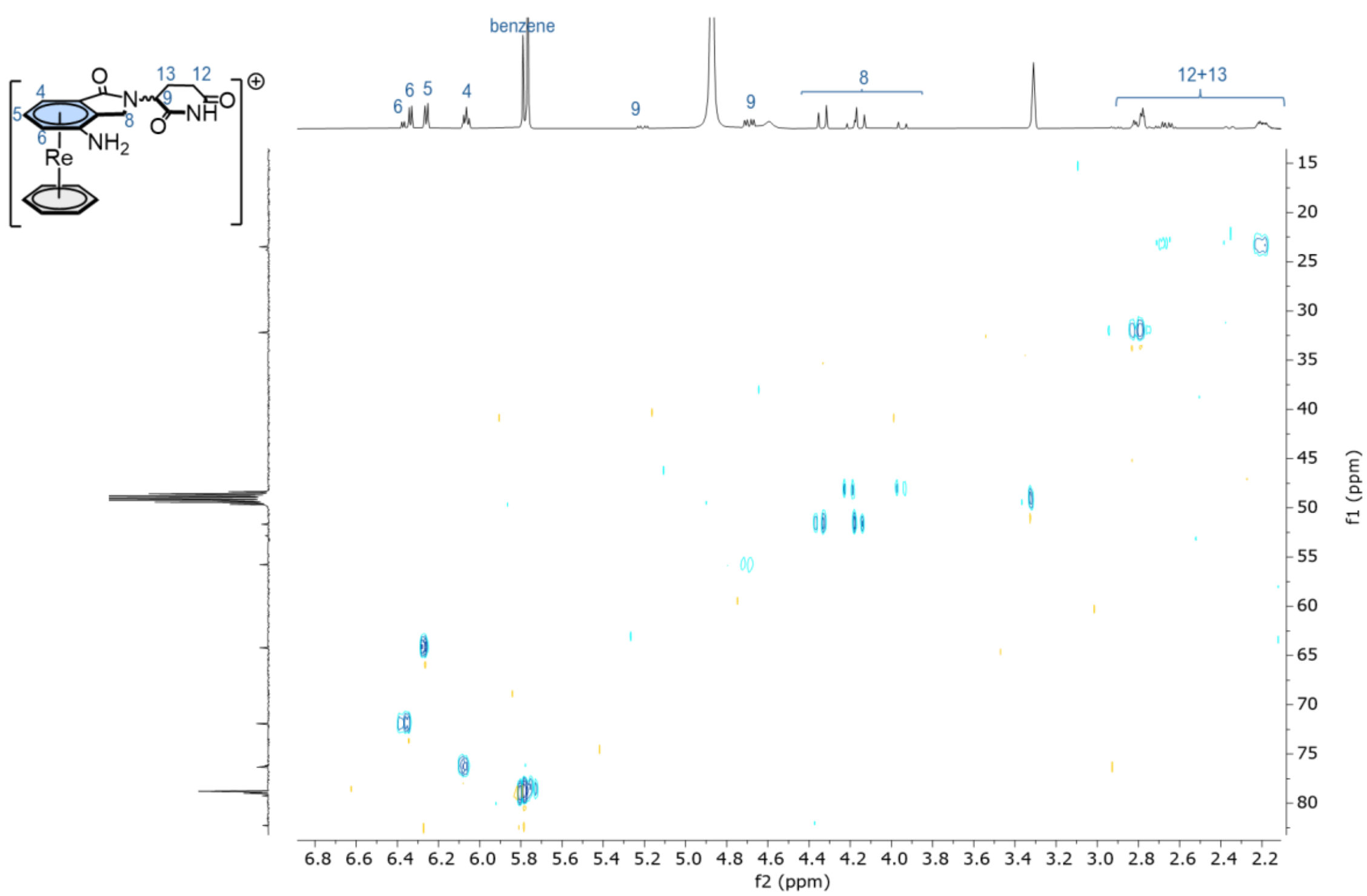

Figure S13. ^{1}H-^{13}C HSQC NMR spectrum in CD_3OD of [Re(η⁶-benzene)(η⁶-lenalidomide)]$^{+}$ (**2**), both diastereoisomers.

X-ray crystalography

Table S1. Crystal data and data collection of [Re(η[6]-lena)$_2$]$^+$ (**1**) of fraction B, enantiomeric pair (**U**,*S*; **U**,*R*) and (**D**,*R*; **D**,*S*) and fraction C, enantiomeric pair (**U**,*R*; **U**,*R*) and (**D**,*S*; **D**,*S*)

	(**U**,*S*; **U**,*R*)/(**D**,*R*; **D**,*S*)	(**U**,*R*; **U**,*R*)/(**D**,S; **D**,*S*)
CCDC number	2538046	2538047
Empirical formula	$C_{27.71}H_{27.48}F_{2.57}N_6O_{9.03}Re_{1.15}$	$C_{28}H_{26}F_3N_6O_8Re$
Formula weight	850.98	817.75
Temperature/K	160.0(1)	160.0(1)
Crystal system	orthorhombic	triclinic
Space group	$Pca2_1$	P-1
a/Å	16.3189(2)	6.96382(19)
b/Å	12.7907(2)	10.9633(3)
c/Å	13.8647(2)	21.4702(4)
α/°	90	89.6779(18)
β/°	90	87.8035(19)
γ/°	90	72.419(3)
Volume/Å^3	2893.98(8)	1561.43(7)
Z	4	2
ρ_{calc}g/cm^3	1.953	1.739
μ/mm^{-1}	10.099	8.287
F(000)	1668.0	804.0
Crystal size/mm^3	0.06 × 0.03 × 0.01	0.05 × 0.04 × 0.03
Radiation	Cu Kα (λ = 1.54184)	Cu Kα (λ = 1.54184)
2Θ range for data collection/°	8.784 to 136.406	8.242 to 136.492
Index ranges	$-19 \leq h \leq 14, -15 \leq k \leq 15, -16 \leq l \leq 16$	$-8 \leq h \leq 8, -13 \leq k \leq 13, -25 \leq l \leq 25$
Reflections collected	43086	31782
Independent reflections	5275 [R_{int} = 0.0704, R_{sigma} = 0.0411]	5623 [R_{int} = 0.0384, R_{sigma} = 0.0269]
Data/restraints/parameters	5275/571/474	5623/327/481
Goodness-of-fit on F^2	1.057	1.081
Final R indexes [I>=2σ (I)]	R_1 = 0.0392, wR_2 = 0.0990	R_1 = 0.0643, wR_2 = 0.1828
Final R indexes [all data]	R_1 = 0.0444, wR_2 = 0.1021	R_1 = 0.0719, wR_2 = 0.1914
Largest diff. peak/hole / e Å^{-3}	1.33/-1.35	3.42/-1.08
Flack parameter	-0.012(10)	-

Table S2. Crystal data and data collection of $[Re(\eta^6\text{-}C_6H_6)(\eta^6\text{-lena})]^+$ (**2**) enantiomeric pair (**U**,*R*) and (**D**,*S*) and enantiomeric pair (**U**,*S*) and (**D**,*R*)

	(**U**,*R*)/(**D**,*S*)	(**U**,*S*)/(**D**,*R*)
CCDC number	2538048	2538049
Empirical formula	$C_{21}H_{19}F_3N_3O_5Re$	$C_{21}H_{19}F_3N_3O_5Re$
Formula weight	636.59	636.59
Temperature/K	160.0(1)	160.0(1)
Crystal system	monoclinic	monoclinic
Space group	$P2_1/n$	$P2_1$
a/Å	8.9326(2)	8.90428(18)
b/Å	19.2712(5)	21.3753(4)
c/Å	12.5326(3)	11.09928(18)
α/°	90	90
β/°	108.112(3)	92.1307(16)
γ/°	90	90
Volume/Å^3	2050.49(9)	2111.08(7)
Z	4	4
ρ_{calc}g/cm^3	2.062	2.003
μ/mm^{-1}	12.218	11.868
F(000)	1232.0	1232.0
Crystal size/mm^3	0.31 × 0.26 × 0.13	0.12 × 0.08 × 0.03
Radiation	Cu Kα (λ = 1.54184)	Cu Kα (λ = 1.54184)
2Θ range for data collection/°	8.728 to 148.944	7.972 to 152.768
Index ranges	$-11 \le h \le 8$, $-24 \le k \le 23$, $-15 \le l \le 15$	$-11 \le h \le 10$, $-26 \le k \le 26$, $-13 \le l \le 13$
Reflections collected	20423	41607
Independent reflections	4175 [R_{int} = 0.0359, R_{sigma} = 0.0173]	8751 [R_{int} = 0.0267, R_{sigma} = 0.0189]
Data/restraints/parameters	4175/516/372	8751/1508/725
Goodness-of-fit on F^2	1.165	1.059
Final R indexes [I>=2σ (I)]	R_1 = 0.0498, wR_2 = 0.1100	R_1 = 0.0369, wR_2 = 0.0988
Final R indexes [all data]	R_1 = 0.0501, wR_2 = 0.1102	R_1 = 0.0378, wR_2 = 0.0997
Largest diff. peak/hole / e Å^{-3}	2.43/-1.44	1.66/-1.20
Flack parameter	-	0.50(2)

In Vitro Cellular Binding of [Re(η^6-lena)$_2$]$^+$ (1) and [Re(η^6-C$_6$H$_6$)(η^6-lena)]$^+$ (2)

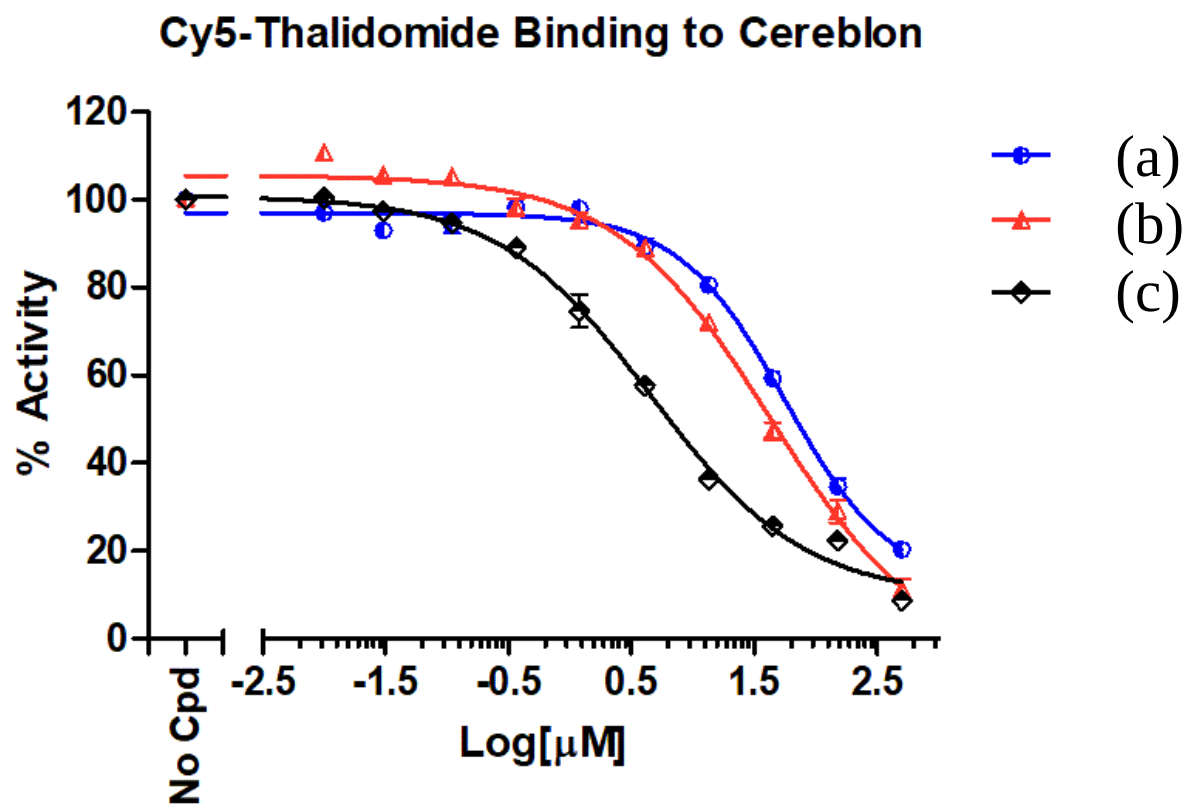

Figure S14. *In vitro* Cy5-Thalidomide binding to CRBN. Changes in fluorescence polarization are measured at different concentrations of a) [Re(η^6-C$_6$H$_6$)(η^6-lena)]$^+$ (2), b) [Re(η^6-lena)$_2$]$^+$ (1) and c) Lenalidomide, which are used as competing ligands to determine IC$_{50}$ values. Data is presented as average of three replicates ± SD

Table S3. Summary of inhibitory effects of all tested compounds on CRBN activity

**Compound**	**IC$_{50}$ (µM)**	**95% C.I. (µM)**
[Re(η^6-C$_6$H$_6$)(η^6-lena)]TFA	~74	61.2 to 85.5
[Re(η^6-lena)$_2$]TFA	43.8	35.6 to 52.5
Lenalidomide	7.6	4.9 to 9.6
Pomalidomide	3.0	2.7 to 3.4

Table S4. Inhibitory effects of [Re(η^6-C$_6$H$_6$)(η^6-lena)]TFA (2TFA) on CRBN activity.

**[Re(lenalidomide)(benzene)]TFA**	**CRBN Binding [(Fluorescent Polarization (mp)]**			**% Activity**		
	Repeat1	Repeat2	Repeat3	Repeat1	Repeat2	Repeat 3
No Compound	83	82	82	101	100	99
0.01 µM	80	81	81	96	97	98
0.03 µM	77	80	78	91	96	92
0.11 µM	78	78	81	92	93	98
0.36 µM	81	82	82	97	99	99
1.2 µM	81	81	82	98	97	99
4.05 µM	78	75	75	93	87	88
13.5 µM	71	71	72	81	80	81
45 µM	60	56	59	61	56	61
150 µM *	43	42	45	34	32	38
500 µM *	36	34	36	21	19	21
Background	25	23	21			

Table S5. Inhibitory effects of [Re(η^6-lena)$_2$]TFA (1TFA) on CRBN activity.

**[Re(lenalidomide)$_2$] TFA**	**CRBN Binding [(Fluorescent Polarization (mp)]**			**% Activity**		
	Repeat1	Repeat2	Repeat3	Repeat1	Repeat2	Repeat 3
No Compound	75	75	76	100	100	101
0.01 µM	82	81	81	112	110	110
0.03 µM	79	79	78	106	106	105
0.11 µM	78	78	80	104	104	108
0.36 µM	74	77	73	97	102	96
1.2 µM	72	75	73	93	99	94
4.05 µM	69	71	69	88	92	87
13.5 µM	60	60	62	71	70	75
45 µM	48	46	50	47	44	51
150 µM *	37	41	37	27	34	26
500 µM *	28	28	31	9	9	16
Background	25	23	21			

Table S6. Inhibitory effects of Lenalidomide on CRBN activity.

**Lenalidomide**	**CRBN Binding [(Fluorescent Polarization (mp)]**			**% Activity**		
	Repeat1	Repeat2	Repeat3	Repeat1	Repeat2	Repeat 3
No Compound	84	83	86	100	98	102
0.01 µM	85	84	84	102	100	99
0.03 µM	82	83	84	95	98	99
0.11 µM	80	82	81	93	97	94
0.36 µM	76	79	78	86	91	90
1.2 µM	64	71	72	67	78	79
4.05 µM	58	58	59	57	58	58
13.5 µM	47	45	44	39	36	34
45 µM	39	40	38	26	27	24
150 µM	36	36	38	21	21	25
500 µM	27	29	28	7	10	9
Background	25	23	21			

Table S7. Inhibitory effects of Pomalidomide on CRBN activity.

**Pomalidomide**	**CRBN Binding [(Fluorescent Polarization (mp)]**			**% Activity**		
	Repeat1	Repeat2	Repeat3	Repeat1	Repeat2	Repeat 3
No Compound	81	80	81	100	99	100
0.01 µM	80	80	83	100	99	105
0.03 µM	79	82	76	97	102	90
0.11 µM	78	78	75	95	94	88
0.36 µM	76	76	74	91	90	87
1.2 µM	68	65	68	72	66	71
4.05 µM	56	56	55	45	45	43
13.5 µM	45	44	46	21	19	23
45 µM	37	34	36	2	0	1
Background	19	21	23			

***In vivo* biodistribution studies of $[^{99m}Tc(\eta^6\text{-lena})_2]^+$ (3)**

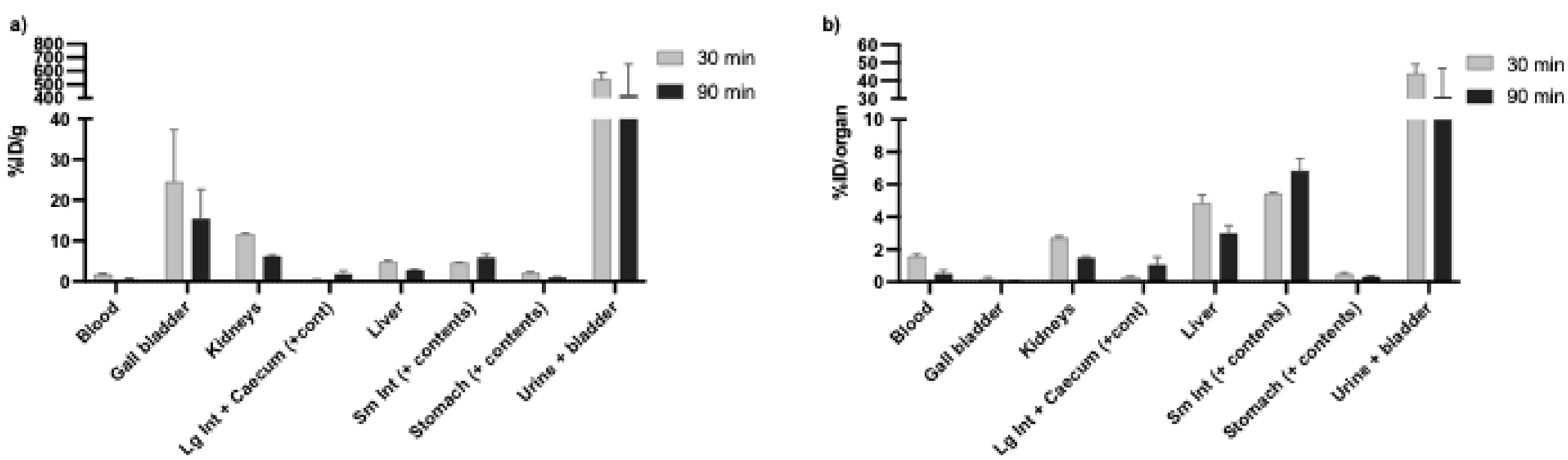

Figure S15. Biodistribution studies of $[^{99m}Tc(lena)_2]^+$ (3) in Balb/c mice. Two time points were evaluated, 30 and 90min (n = 3). a) %ID/g, b) %ID/o. Data is presented as average ± SEM.

Table S8. Biodistribution data for $[^{99m}Tc(\eta^6\text{-lena})_2]^+$ (3) expressed as percent injected dose per gram (%ID/g). Data were taken at the indicated timepoints, and studies were performed in Balb/c mice.

**%ID/g**	**30min n=3**		**90min n=3**	
**Organs**	**Avg**	**SEM**	**Avg**	**SEM**
**Blood**	1.71	0.14	0.52	0.26
**Adipose**	0.24	0.06	0.15	0.03
**Adrenals**	0.70	0.05	0.34	0.01

**Bone**	0.54	0.03	0.13	0.00
**Brain**	0.07	0.01	0.03	0.00
**Gall bladder**	24.49	12.81	15.47	7.10
**Heart**	0.55	0.02	0.13	0.01
**Kidneys**	11.55	0.30	6.16	0.35
**Lg Int + Caecum (+cont)**	0.49	0.03	1.75	0.88
**Liver**	4.81	0.36	2.82	0.32
**Lungs**	1.31	0.13	0.27	0.03
**Pancreas**	0.47	0.05	0.36	0.12
**Skeletal Muscle**	0.35	0.02	0.07	0.01
**SmInt (+ contents)**	4.64	0.15	5.88	0.83
**Spleen**	0.52	0.01	0.19	0.01
**Stomach (+ contents)**	2.05	0.32	1.02	0.09
**Thyroid/Trachea**	1.45	0.12	0.56	0.07
**Urine + bladder**	537.45	51.45	420.95	228.35

Table S9. Biodistribution data for $[^{99m}Tc(\eta^6\text{-lena})_2]^+$ (3) expressed as percent injected dose per organ (%ID/o). Data were taken at the indicated timepoints, and studies were performed in Balb/c mice.

**%ID/O**	**30min n=3**		**90min n=3**	
**Organs**	**Avg**	**SEM**	**Avg**	**SEM**
**Blood**	1.57	0.14	0.48	0.24
**Adipose**	0.02	0.00	0.01	0.00
**Adrenals**	0.01	0.00	0.01	0.00
**Bone**	1.08	0.08	0.26	0.01
**Brain**	0.03	0.00	0.01	0.00
**Gall bladder**	0.16	0.13	0.06	0.03
**Heart**	0.05	0.00	0.01	0.00
**Kidneys**	2.72	0.10	1.48	0.06
**Lg Int + Caecum (+cont)**	0.30	0.02	1.04	0.50
**Liver**	4.85	0.50	2.98	0.49
**Lungs**	0.15	0.03	0.03	0.00
**Pancreas**	0.06	0.01	0.05	0.02
**Skeletal Muscle**	2.53	0.14	0.49	0.07
**SmInt (+ contents)**	5.44	0.10	6.83	0.78
**Spleen**	0.04	0.00	0.02	0.00
**Stomach (+ contents)**	0.47	0.09	0.32	0.03
**Thyroid/Trachea**	0.03	0.00	0.02	0.00
**Urine + bladder**	43.82	5.21	31.21	15.65

References

[27] R. C. Clark, and J. S. Reid, "The Analytical Calculation of Absorption in Multifaceted Crystals*," Acta Crystallographica Section A Foundations of Crystallography* 51 *(***1995**): 887-897.

[28] CrysAlisPro (Rigaku Oxford Diffraction Ltd, **2026**).

[29] O. V. Dolomanov, L. J. Bourhis, R. J. Gildea, J. A. K. Howard, and H. Puschmann, "*OLEX2*: A Complete Structure Solution, Refinement and Analysis Program," *Journal of Applied Crystallography* 42 *(***2009**): 3S39-341.

[30] G. M. Sheldrick, "*SHELXT* – Integrated Space-Group and Crystal-Structure Determination," *Foundations of Crystallography* 71 *(***2015**): 3-8.

[31] G. M. Sheldrick, "*Crystal Structure* Refinement With SHELXL," *Crystal Structure Communications* 71 *(***2015**): 3-8.

[32] A. L. Spek, "Structure Validation in Chemical Crystallography," *Biological crystallography* 65 *(***2009**): 148-155.
